# Enabling urban systems transformations: co-developing national and local strategies

**DOI:** 10.1186/s42854-023-00049-9

**Published:** 2023-02-20

**Authors:** Robert Webb, Tayanah O’Donnell, Kate Auty, Xuemei Bai, Guy Barnett, Robert Costanza, Jago Dodson, Peter Newman, Peter Newton, Eleanor Robson, Chris Ryan, Mark Stafford Smith

**Affiliations:** 1 Institute for Climate, Energy and Disaster Solutions (ICEDS) and Fenner School of Environment and Society, Australian National University, HC Coombs Building, 9 Fellows Road, Acton, Canberra, ACT 2601 Australia; 2 Institute for Climate, Energy and Disaster Solutions (ICEDS) and Fenner School of Environment and Society, Australian National University, Building 141, Linnaeus Way, Acton, Canberra, ACT 2601 Australia; 3 University of Melbourne, 21-23 Railway Street, Euroa, VIC 3666 Australia; 4 Fenner School of Environment & Society, Australian National University, Frank Fenner Building, Linnaeus Way, Canberra, ACT 2601 Australia; 5 CSIRO Environment, Commonwealth Scientific and Industrial Research Organisation (CSIRO), Clunies Ross Street, Canberra, ACT 2601 Australia; 6 Institute for Global Prosperity, University College London, Floor 7, Maple House, 149 Tottenham Court Road, London, W1T 7NF UK; 7 Centre for Urban Research, RMIT University, PO Box 2476, Melbourne, VIC 3000 Australia; 8 CUSP, Curtin University, Kent Street, Bentley, Perth, WA 6845 Australia; 9 Centre for Urban Transitions, Swinburne University of Technology, EW Building, Serpells Lane, Hawthorn, Melbourne, VIC 3122 Australia; 10 Future Earth Australia, Ian Potter House, 9 Gordon Street, Acton, Canberra, ACT 2601 Australia; 11 School of Design, RMIT University, GPO Box 2476, Melbourne, VIC 3001 Australia; 12 CSIRO Environment, Commonwealth Scientific and Industrial Research Organisation (CSIRO), PO Box 1700, Canberra, ACT 2601 Australia

**Keywords:** Urban systems, Cities, Transdisciplinary, Transformation, Enablers, Cumulative knowledge, Learning, Power

## Abstract

Transformative urban development is urgent to achieve future sustainable development and wellbeing. Transformation can benefit from shared and cumulative learning on strategies to guide urban development across local to national scales, while also reflecting the complex emergent nature of urban systems, and the need for context-specific and place-based solutions. The article addresses this challenge, drawing on extensive transdisciplinary engagement and National Strategy co-development processes for Australia. This includes generation of two frameworks as boundary objects to assist such transdisciplinary strategy development. An ‘enabling urban systems transformation’ framework comprises four generic overarching transformation enablers and a set of necessary underpinning urban capacities. This also built cumulatively on other sustainability and urban transformation studies. A complementary ‘knowledge for urban systems transformation’ framework comprises key knowledge themes that can support an integrated systems approach to mission-focused urban transformations, such as decarbonising cities. The article provides insights on the transdisciplinary processes, urban systems frameworks, and scoping of key strategies that may help those developing transformation strategies from local to national scales.

**Science highlights**

• Transdisciplinary national urban strategy development is used to distil generic frameworks and strategy scopes with potential international application.

• The frameworks also build on other published framings to support convergent, cumulative and transdisciplinary urban science.

• The ‘enabling transformations’ and ‘urban knowledge’ frameworks include the perspective of those developing sustainable urban systems strategies.

• The enabling framework also informs ‘National Urban Policy’ and ‘Knowledge and Innovation Hub’ strategies, and prevailing power imbalances.

• The knowledge framework can help frame urban challenges, missions and knowledge programs.

**Policy and practice recommendations**

• An urban ‘transformation imperative’ and ‘strategic response’ can be co-developed from local to national scales.

• Local initiative is crucial to drive urban strategies, but sustained national leadership with coherent policy across sectors and scales is also key.

• Diversity in engagement participation and processes generates whole-of-urban-systems and local-to-national perspectives.

• Urban solutions are context-specific but generic frameworks can help collaborative issue framing and responses.

• Collaborative issue framing informed by generic frameworks can bring broader perspectives to context-specific and contested policy and practice issues.

**Supplementary Information:**

The online version contains supplementary material available at 10.1186/s42854-023-00049-9.

## Introduction

Well-managed urbanisation is increasingly critical to achieving national and global Sustainable Development Goals (SDGs) (UN 2015). The percentage of the world’s population living in urban areas is projected to increase from 55 to 68% by 2050, adding 2.5 billion people to the urban population (UNDESA 2019). Urban outcomes significantly impact all 17 of the SDGs (UCLG 2016) and related global commitments on climate change (UNFCCC 2015 Paris Agreement), disaster risk reduction (UNDRR 2015 Sendai Framework) and biodiversity (UNCBD 2022 Global Biodiversity Framework), as well as social equity and many other urban issues (UN-Habitat 2016, 2020). Addressing such challenges concurrently and with increased urgency will require translation of SDGs to local scales (OECD 2020) and relatively rapid transformational changes to urban systems, processes and outcomes.

Cities can be understood as complex and emergent social-ecological-technological systems (SETS) with the understanding that ‘social’ also includes cultural, economic and governance, ‘ecological’ includes climate and biophysical, and ‘technological’ includes engineered and built environment dimensions (Zhou et al. 2021; McPhearson et al. 2022). These are interconnected across sectors and scales (local to international), providing a challenge to siloed and spatially uncoordinated decision-making (Bai et al. 2016a). Given the diversity of local histories, cultures and contexts, specific urban solutions also need to be context-specific and place-based (Corburn 2009; Dixon and Tewdwr-Jones 2021), and guided by local communities’ shared visions and exploration of future pathways (Bai et al. 2016b; McPhearson et al. 2017; Hajer and Versteeg 2019). The complex, systemic and cross-scale nature of urban challenges means that transformative change requires both top-down (national/state) and bottom-up strategies (Ehnert et al. 2018; Romero-Lankao et al. 2018), with solution-oriented transdisciplinary engagement (McPhearson et al. 2022). A national urban systems transformation strategy, co-developed with local-to-national scale and cross-sector stakeholders, can therefore be an important step forward.

Notwithstanding the multi-scale complexity and diversity there is also understandable interest in interdisciplinary convergence and cumulative knowledge-building in urban science (Ramaswami et al. 2018; Acuto et al. 2018; Bettencourt 2021; Zhou et al. 2021) and broader sustainability science (Irwin et al. 2018; Pauliuk 2020; Newig and Rose 2020). This includes development of boundary objects (Mollinga 2008), such as integrating frameworks, to progress shared understanding in complex interdisciplinary and transdisciplinary settings (Lang et al. 2012), and cumulative knowledge-building (Ostrom 2009). Such frameworks could support more systematic approaches to developing urban solutions (Lin et al. 2021) and transferable knowledge for quicker and concerted effort at scale (Bai et al. 2010; Simon et al. 2018).

This article addresses the above challenges drawing on the experience of co-developing a national strategy to enable urban systems transformation for Australian cities and settlements via extensive transdisciplinary processes. Australia’s population has almost tripled since 1950 with a high proportion in urban areas (86% in 2018, projected 91% by 2050 (UNDESA 2019)). The population is growing faster than most other developed countries, projected from 26 m people in 2020 to around 39 m by 2060 (CoA 2021). Effective urban development is therefore crucial for Australia’s sustainable development, and ability to play its part in meeting international challenges and commitments. Australia has exhibited episodic national policy interest in cities since the end of WWII (Dodson 2013), but with some greater continuity since 2007. This included publication of a national urban policy in 2011 and use of federal investment to shape selected urban developments, with however, no overarching urban research strategy and limited research linkage to policy development.

An Australian Urban Systems Transformation (AUST) co-design initiative was carried out to frame Australia’s urban issues (Webb et al. 2018). The initiative was adopted in early 2018 by Future Earth Australia (FEA: the national node in the global Future Earth network), and its host organisation the Australian Academy of Science, as a first significant priority for FEA (FEA 2021). FEA then coordinated development of a National Strategy to enable urban systems transformation, with the intent of also contributing to international knowledge and action.

Drawing on the National Strategy co-development this article contributes to urban sustainability science by identifying transdisciplinary and cumulative approaches with relevance across different local-to-national urban contexts. Specifically, the article:

- *Provides insights on transdisciplinary approaches that can be used to develop transformation strategies for complex urban systems, and related enablers, from local-to-national scales (Section “**Reflections on the co-development process**”).* In this context ‘transformation’ means the changes needed to address a significant gap between longer-term societal aspirations and the current status, typically requiring simultaneous change across multiple interdependent urban sub-systems (for example across land use, transport, energy and environmental systems to achieve interlinked outcomes); and ‘enablers’ refers to the underpinning urban capacities necessary to support multiple such transformations.
- *Presents a cumulative ‘enabling urban systems transformation’ framework, and a complementary ‘knowledge’ framework, that together can operate as boundary objects to assist transdisciplinary urban engagement, and holistic urban strategy, mission and knowledge framing and development (Sections “**The EUST and KUST frameworks**”/ “**Systems-wide frameworks as boundary objects**”)*.
- *Provides insights on urban transformation strategies, including an approach to scoping key top-down and bottom-up strategies (*i.e. *National Urban Policy and local Knowledge and Innovation Hubs) to be complementary and fit-for-purpose (Sections “**Linking the frameworks to strategies**"/ "**Some high leverage enabling strategies**”).*

## The co-development methodology and analysis processes

**Fig. 1:**
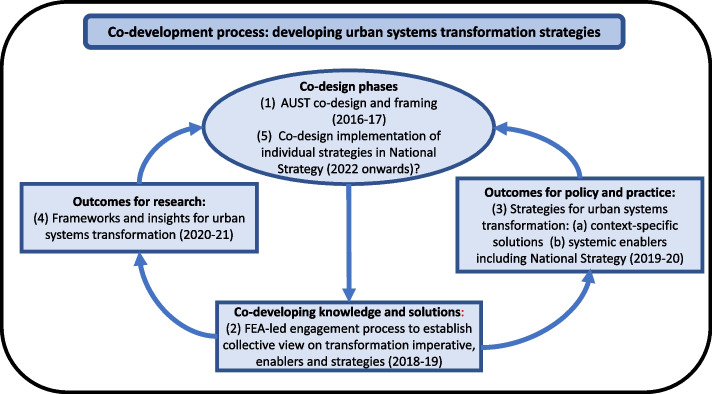
Enabling urban systems transformation: the co-development process. The process shows the stages in co-developing ‘urban systems transformation’ strategies. Stage (1), the Australian Urban Systems Transformation (AUST) co-design and framing initiative, was covered in Webb et al. (2018) and the overall process is consistent with the methodology therein. The current article covers Stages (2), (3), (4). Stage (5) is the proposed next step

The Australian urban systems transformation strategy co-development process followed is shown in Fig. 1. This article covers Stages (2), (3), (4) in this process. Stage (5) is proposed for the future. The earlier Australian Urban Systems Transformation (AUST) co-design and framing work (Stage (1)) had found that key transformation enablers seemed inadequate to the challenge across all scales (Webb et al. 2018). Collective urban visioning and goal development was scarce; stakeholder and community engagement by decision-makers was limited and often appeared tokenistic; institutional, governance and decision-making coherence was lacking; and urban knowledge development, while excellent in many respects, was fragmented, with uptake well below potential. These findings needed to be tested and extended with a broader range of stakeholders and researchers, with a view to developing a strategic response.

### The National Strategy co-development process

The National Strategy co-development process (hereafter the ‘FEA process’) was coordinated by FEA between May 2018 and December 2019 (Stages (2), (3) in Fig. 1). The first step was a May 2018 National Symposium (FEA 2018) of (largely) researchers, including representatives of all major urban research programs in Australia, supplemented by interviews with individual researchers. There was a broad consensus from the Symposium on the framing diagnosis, and that FEA further test this with stakeholders and coordinate the co-development of a National Strategy across scales (local-city-region-state/territory-national).

FEA, supported by a network of researchers from Australia’s states and territories, (including the authors of this article), led an extensive transdisciplinary engagement process. This recognised that ‘urban’ is about smaller regional cities and settlements as well as major cities, and the connections these have locally (including to rural hinterlands), nationally and internationally. The objective was to establish the views of diverse urban stakeholders and communities on their aspirations for Australian urban cities and settlements of the future, current issues being experienced, and the strategies to move towards their aspirations. Stakeholders represented included federal, state/territory and local governments, utilities, the urban professions, private sector, social and environmental non-government organisations (NGOs), community representatives, and academia. An inclusive approach was adopted to understand the widest possible variety of urban experience and perspectives. Activities included:

- creation of a cross-discipline and cross-sector Urban Reference Group to help guide the process;
- multi-stakeholder workshops held in each state and territory capital city except Hobart (i.e. Brisbane, Sydney (two), Canberra, Melbourne, Adelaide, Perth, Darwin), plus Alice Springs, with individual workshop outcome reports produced (FEA 2019). Altogether there were approximately 400 participants, with local representation of the full range of stakeholder sectors noted above. To reflect the voices of the most marginalised peoples, this included for example local participants from the Australian Council of Social Services, a body representing thousands of front-line community agencies that advocates to reduce poverty and inequality, and for social justice for First Nations’ people. Extra perspective directly from First Nations’ communities came from the workshops in Darwin and Alice Springs and an indigenous ‘yarning circle’ in Darwin;
- forty semi-structured interviews with key stakeholders at all levels, but with an emphasis on the national level, including government departments/agencies, professions’ peak bodies, industry/business peak bodies, and NGOs; and additional interviews with individual researchers;
- written submissions invited from stakeholders;
- a citizen survey, initially for self-selected responders (see Costanza-van den Belt et al. (2021) for the process and outcomes), but with a view to future extension to other cohorts and a nationally representative random sample of citizens;
- review of literature, reports and websites on Australian urban development, planning, and research;
- based on the above inputs, iterative preparation of the National Strategy coordinated by FEA, with the progressive guidance of the Urban Reference Group, and input on exposure drafts from the wider stakeholder networks, roundtables, workshop and interview participants before finalisation; and
- engagement with key politicians and policy-makers during 2020 to discuss and promote the National Strategy proposals; and with other stakeholders with potential support roles.

The workshops and interviews, which provided the majority of direct stakeholder input, were deliberately unstructured except for broad facilitative framing of the objectives. As no national exercise like this had been conducted before on urban transformation, it was important to not constrain or pre-empt the issues and ideas raised, and this was rewarded with a rich range of insights. A synthesis of the key themes, issues and opportunities identified was prepared (Webb and O’Donnell 2019). The above processes provided the basis for the published National Strategy, a ‘Ten-year strategy to enable urban systems transformation’ for more sustainable cities and regions (O’Donnell et al. 2019).

### Analysis for broader insights

The extensive input from the strategy co-development process provided an opportunity to identify broader implications for urban systems transformation approaches, both nationally and internationally (Stage (4) in Fig. 1). For this purpose, the following additional analyses have been carried out by the authors:

1. The detailed inputs from participants were re-analysed to
  clarify the validity, nature and scope of the overarching urban systems transformation enablers that had been provisionally identified from the earlier co-design and framing work (i.e. shared visioning, stakeholder/community engagement, institutional coherence, and knowledge development and use); and
  identify the generic transformation capacities that underpin each of these enablers, for possible application to other urban contexts.
2. The National Strategy’s proposed actions were mapped to the overarching enablers and underpinning capacities they support, to seek insights on urban strategy development.
3. In parallel with the above analyses, the findings were compared with international literature on sustainability and urban transformation frameworks to assess the potential for more generic ‘urban systems transformation’ frameworks.

## Results: Responding to urban transformation imperatives

“The transformation imperative” section summarises the results from the FEA process (Stages (2), (3) in Fig. 1), which identified a number of issues that evidenced an urban transformation imperative for Australia (see Webb and O’Donnell et al. (2019) for more detail) and in response the proposed National Strategy. This is followed by sections that describe the more generic frameworks based on the subsequent analyses (Stage (4) in Fig. 1).

### The transformation imperative

#### Visioning urban futures

In a visioning process in each workshop, participants expressed their aspirations for their cities. These were broadly compatible in scope with overarching frameworks like the SDGs and Quadruple Bottom Line outcomes, yet far from their current lived experience. Issues raised included growing social injustice (e.g. inequitable access to affordable housing, jobs, social infrastructure and green space); urban sprawl but also poorly designed densification; transport disruption, access and congestion; growing impacts of climate change; and loss of urban environmental quality and biodiversity. There were common themes across cities, but also important differences in emphasis, priorities and possible pathways, (reflecting the heterogenous character, strengths, and challenges of different Australian cities), and some potential tensions in the future visions. Additional file 1 provides further detail on current issues and 2030-50 visions identified in the workshops.

The feedback from the FEA process, including workshops, was that these issues required both spatial planning and urban process interventions, many of which resonated with recent proposals from peak industry organisations, urban researchers, and other bodies, but were only partially evident in state and territory government policies, and hardly at all at the federal level. Spatial planning suggestions included more coherent national, state, regional and city settlement planning to balance growth between cities and regions (e.g. PIA 2018); planning within cities to reflect the very different issues for inner, middle and outer suburbs, and improved place-based design including public spaces and blue/green infrastructure (Newton et al. 2022); and integrated land-use and transport planning that reflects different urban fabrics (Newman et al. 2016) and greater proximity between housing, work and services.

Urban process suggestions included more attention to sustainable consumption and production, circular economies, decarbonisation and regeneration strategies, all in order to reduce resource use, waste and pollution (including greenhouse gas emissions). It was also clear that there are many interdependencies between the spatial and process strategies and outcomes (e.g. Thomson and Newman 2018).

Addressing such interdependent and challenging issues was seen as needing a higher level of innovation at all scales - large scale urban infrastructure (IA 2021) as well as local place-based innovations. This might draw on new technologies,^1^ but was also about socio-economic and institutional redesign and innovation to ensure that social justice and equity remain in focus alongside economic and environmental outcomes. A ‘whole-of-urban-systems’ approach was seen as necessary to capture the many synergies and trade-offs embedded in the above issues (e.g. consistency across goals and targets; interdependencies between spatial planning and urban processes; nexus issues between urban sub-systems; framing of issues and business cases to include wider economic and non-economic costs and benefits).

The urban transformation imperative was reinforced by the government-funded Australian National Outlook (CSIRO 2019) which concluded that sustainable urban development is one of five major shifts Australia needs to undergo to move to a more desirable future trajectory. The other four shifts were industrial composition, energy, land use and culture – each also highly relevant in the urban context. Nationally, Australia has challenges in meeting many of the SDGs (Allen et al. 2019; MSDI 2020) and more sustainable urban development will be critical. These and other urban issues identified above ultimately require context-specific solutions, but the FEA process participants also identified more systemic barriers and enablers.

#### Systemic transformation barriers and enablers

##### Navigating towards an emergent future

Examples were cited of Australian scenario-based planning approaches to support participative future visioning and pathways (e.g. scenarios for settlement strategies with alternative land use, transport and densification approaches (CSIRO 2019; IA 2019); low-carbon-living visions and place-based co-design (Ryan et al. 2016; Candy et al. 2017); alternative governance and community values assumptions (Moglia et al. 2018); and to assess Australia’s alignment with the SDGs (Allen et al. 2019)). There was also a desire to link urban planning to local translation of the SDGs, with a clear line-of-sight between levels, and better evidence to assess trade-offs and synergies in goals and target setting. Participants sought to improve the content and utility of the current National Cities Performance Framework (BITRE 2021) with translation to all levels as a basis for monitoring and strategy adjustment. Especially strong support emerged for creation of local or city-based ‘Knowledge and Innovation Hubs’ or equivalent, as vehicles for facilitating local initiatives, experimenting, learning and sharing knowledge. Many initiatives were mentioned as good examples or opportunities, but there was no systematic approach to developing, upscaling and sharing the innovations and learning to influence broader strategies.

None of the above approaches were yet mainstreamed into government-led urban planning and decision-making, which were seen as overly influenced by short-term political and developer interests, or outdated government regulations and practices, rather than visionary and collaborative strategies; and in any event subject to poor and distorted implementation. It was recognised that without each of the above strategic components working together it would not be possible to navigate towards a shared, intentional but also emergent future.

##### Decision-makers engaging with stakeholders and communities

There were examples noted of good and bad engagement practices, from national to local levels, and recognition of the value of diverse knowledge sources, including marginalised and First Nation’s peoples (since reinforced by the FEA-coordinated *National Strategy for Just Adaptation* (FEA 2022)). The diversity of engagement contexts was recognised, including purposes along the engagement spectrum from simple information-sharing to developing local empowerment (IAP2 2018). Participants sought more inclusive and meaningful engagement at all levels, with a progressive move to more collaborative approaches and techniques, but tailored to best practices (which needed to be developed and promulgated) for the issues at hand. Engagement with stakeholders and communities was seen as important to better understand lived experience, the rationale behind current stakeholder and citizen behaviours, and the values driving future expectations.

##### Institutions and governance

This generated the most input of all the topics, particularly on government policy, planning, decision-making and resource allocations. Concerns included lack of coherence, both horizontally across agencies and vertically between the federal, state and local governments. Decision-making at all levels was seen as lacking in rigour and transparency (Grattan Institute 2021), and too often influenced by short term political and private sector interests rather than being evidence-based. Federal governments have run urban policy with little continuity over time, considering it mostly a state government matter despite its implications for achieving national outcomes such as emissions reductions, well-being and the SDGs. State governments prepare the larger city-region plans, and the next level of formal governance for most matters is the local government or council. The capital cities generally have multiple councils (Sydney and Melbourne around 30-40), which individually are not well resourced (Productivity Commission 2011). Lack of a metropolitan level of governance adds to the fragmentation (Tomlinson and Spiller 2018). There were some signs of change, for example Sydney introducing the Greater Cities Commission to focus on Sydney and region strategic planning (GCC 2022), metropolitan-scale resilience strategies developed for Melbourne and Sydney, and the federal government initiating City/Regional Deals to coordinate across the three levels of government on a set of agreed initiatives in specific locations (CoA 2022). Uniquely in Australia the Australian Capital Territory Government combines most of the powers of state and local government which has enabled decarbonisation (Mummery 2021) and other sustainable development leadership and action. However, participants were seeking stronger and more coherent, transparent and consistent leadership at all levels, and especially from future federal governments (HoR 2018).

##### Knowledge co-production, usage and learning

A large number of relevant knowledge themes were identified, and much excellent research on specific urban topics was evident, often co-produced with stakeholders and communities, and by a number of research bodies.^2^ Most of these have strong international connections, and Australian cities have also been active in global network initiatives (e.g. C40, Rockefeller 100 Resilient Cities, ICLEI). Nevertheless, a key challenge identified was the need for an overarching national urban research strategy linked to policy development and across research institutions. This could increase collaborative and whole-of-urban-systems capacities and knowledge, currently challenged by fragmented governance and research funding, leading to competition rather than collaboration. Also, the uptake of both new and existing knowledge in policy and practice was well below potential, reflecting the need to strengthen mutual understanding and learning between researchers, policy-makers and practitioners, including collaborative development of research agendas and issue framing, and improved synthesis, accessibility, translation and brokering of knowledge.

#### Strategic responses to the transformation imperative

The above FEA process findings have implications for urban policy and practice at two levels (Stages 3(a) and 3(b) in Fig. 1). First, strategic responses will be necessary to address a range of specific transformation challenges and associated missions (Mazzucato 2018; e.g. increased urban use of renewable energy), in order to develop *context-specific solutions* that move towards desired urban futures.

Second, there is a need to develop the urban capacities that will *systemically enable* such transformations. This was the focus of the National Strategy (O’Donnell et al. 2019), and the proposed strategies and actions are summarised in Table 1 (Cols. 1, 2). The Strategy focused on systemic enablers that would support urban solutions from local to national scale.

**Table 1:** National Strategy Actions, and transformation capacities to which they could contribute

High level Strategies (Sx) and actions (Sx.x) from the National Strategy	Additional actions from the National Strategy	Transformation capacities (Table 2)
***Strategy 1 Visions for action: coherence to achieve Sustainable Development Goals (SDGs)***		
S1.1 Establish a collaborative visioning framework to prepare a National Urban Policy (NUP)	Cities greater than 50,000 population to establish metropolitan plans	1.1-1.3; 2.1-2.3; 3.1-3.6; 4.1-4.5
S1.2 Embed the SDGs across all actions and related policy within this National Strategy and the NUP	The NUP also reflects the UN Habitat Program principles for NUPs	1.2; 3.1
S1.3 Align the existing National Cities Performance Framework with the NUP	Goals, targets and performance audit for all cities aligned with the NUP	1.1-1.2; 3.1; 4.5
S1.4 Build knowledge of interactions and trade-offs within urban and regional systems to support national strategy implementation	National urban systems research program established; State of Australian Cities and Regions assessment framework to report on conditions and dynamics in Australian cities and regions; Provide a national information platform to link knowledge to cross-sectoral urban systems innovation	1.1-1.3; 3.1; 4.3
S1.5 Embed participation, engagement, and co-design between researchers, policy makers, business, and communities in development and implementation of the NUP	Establish new practice guidelines for inclusion of diverse stakeholders in urban policy formulation; Institute national arrangements to enable engaged and participatory urban policy formulation	2.1-2.3; 3.2; 4.2
S1.6 Build a knowledge platform and supporting processes on effective engagement, co-design, and participation practices	Platform to be open access	2.1-2.3
***Strategy 2 Enable innovation: to achieve visions***		
S2.1 Establish a National Urban Forum alongside the biennial State of Australian Cities conference to drive a national agenda-setting process	A National Urban Forum operates as a multi-sector, multi-stakeholder event to coordinate knowledge and innovation co-production and exchange	3.5; 4.1-4.2, 4.4-4.5
S2.2 Establish a network of cross-sector local Knowledge and Innovation (K&I) Hubs at city and regional scales across Australia	K&I Hubs established in all cities and regions; formally linked to each other including via the National Urban Forum and knowledge sharing platforms; wide stakeholder involvement	1.1-1.3; 2.1-2.3; 3.1-3.6; 4.1-4.5
***Strategy 3 Connect knowledge: infrastructure to share knowledge***		
S3.1 Expand an open access data sharing and analytics platform supported by governments and industry/ sector partners for a minimum of 10 years	National open access digital platform(s) for collating, indexing, hosting, and disseminating Australian urban research and policy material; linked to stakeholders via National Urban Forum and NUP processes	3.3-3.5; 4.1, 4.4
S3.2 Link Australian researchers and institutions into global urban research networks	Australian urban researchers and institutions supported to participate in overseas research and policy collaborations; drawing on the K&I Hubs	3.5; 4.1, 4.4
***Strategy 4 Build capacity: of researchers, practitioners and policy-makers***		
S4.1 Build the capacity of urban researchers to engage with policymakers to deliver practicable knowledge linked to the NUP, the SDGs, and K&I Hubs	Training in policy engagement and research-to-policy translation in university teaching and development programs; new trans-disciplinary cross-institutional PhD program linked to the NUP; nationally funded secondments to, and research with, policy and practice organisations	4.4
S4.2 Build the capacity of urban practitioners to engage with researchers to apply research in policy and practice, linked to the NUP and the SDGs	Scholarships and secondments funded for policy and practice professionals to join research programs aligned to the NUP; professional bodies supported to strengthen practitioner capability and accreditation in commissioning and use of research	3.3; 4.4

Cols. 1, 2 are National Strategy proposed actions summarised from O’Donnell et al. (2019). These actions can develop the transformation capacities in Col. 3 (see Table 2 for ‘Col. 3’ capacity descriptions in the EUST framework)

### The EUST and KUST frameworks 

#### EUST framework: Enabling Urban Systems Transformation

**Fig. 2:**
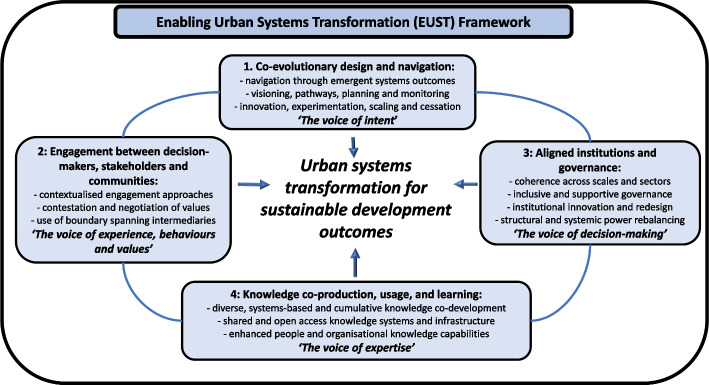
Enabling Urban Systems Transformation (EUST) Framework. Framework of four enablers or ‘voices’ based on the FEA engagement process findings. Urban capacities underpinning each enabler are in Table 2 (more detail at Additional file 2)

Following development and promulgation of the National Strategy through 2019-20, the next step was to distil more generic insights from the FEA process (Stage (4) in Fig. 1). The detailed findings were revisited to crystallise the overarching urban systems transformation enablers (Fig. 2) and themes that identified the urban capacities required to underpin each enabler (Table 2). Together, these constitute an Enabling Urban Systems Transformation (EUST) framework that could have application beyond the Australian context.

**Table 2:** Enabling Urban Systems Transformation (EUST) framework, and underpinning capacities

Four enablers (x) and underpinning capacities (x.x) (*note [Cx] indicates closest capacity match in* Wolfram *(*2016*)* *framework)*			
**1. Co-evolutionary design and navigation** ***– ‘The voice of intent’***	**2. Engagement between decision-makers, stakeholders and communities –** ***‘The voice of experience, behaviours and values’***	**3. Aligned institutions and governance –** ***‘The voice of decision-making’***	**4. Knowledge co-production, usage and learning –** ***‘The voice of expertise’***
**1.1. Co-evolutionary intent, design and navigation** **1.2. Shared visioning, scenarios, goal-setting, pathways, planning and performance** *[C5 Sustainability foresight]* **1.3. Experimentation, innovation, and cessation, recognising the evolutionary phases of introducing the new and ceasing the outdated** *[C6 Experiments, plus innovation and exnovation]*	**2.1. Engagement between decision-makers and diverse stakeholders and communities, for mutual understanding, appreciation, negotiation and collaboration** *[C9 Agency levels, C10 Scale levels]* **2.2. Engagement approaches to be tailored to the context** **2.3. Use of boundary spanning intermediaries (e.g. specific issue-based, researchers, consultants)**	**3.1. Aligned institutions and coherent policies, plans, resource allocations, finance and decisions across scales, sectors and systems** *[C10 Scale levels]* **3.2. Inclusive, transparent and community-centred formal and informal urban institutions and governance** *[C1 Inclusive and multiform urban governance, C9 Agency levels]* **3.3. Critical urban planning capabilities** **3.4. Empowered cities, settlements, communities of practice, community groups and individuals** *[C3 Empowered communities of practice]* **3.5. Institutionally supported innovation and technology facilitation, learning embedding and acceleration** *[C7 Innovation embedding]* **3.6. Transformative formal and informal leadership** *[C2 Transformative leadership]*	**4.1. Co-produced, shared and used knowledge** **4.2. Diverse knowledge sources and disciplines** **4.3. Urban systems awareness, knowledge and cumulative understanding** ***(see KUST framework)*** *[C4 System(s) awareness]* **4.4. Policy-practice-research capabilities and collaborations** **4.5. Reflexivity and learning** *[C8 Reflexivity and learning]*

The four enablers are as per Fig. 2. More detail on the underpinning capacities is at Additional file 2. Some of the capacity descriptors deliberately build on the capacities identified in Wolfram (2016) and Wolfram et al. (2019), which have also been mapped here as closely as possible to encourage ongoing cumulative framework development and cumulative knowledge building on the capacities (see Section “Comparison with other studies on transformation enablers and capacities”)

The four enablers of transformation are similar to those referred to in “The co-development methodology and analysis processes” section but the FEA process and subsequent analysis have significantly clarified the desired nature and scope of each.

The analysis identified 17 significant underpinning capacities mapped to the four enablers (Table 2). The precise definition of each capacity has in several cases drawn on other studies in the interests of cumulative knowledge building (see next section “Comparison with other studies on transformation enablers and capacities”). More detail on individual capacities is at Additional file 2, including some key characteristics to help operationalise the capacity, and a summary of the FEA engagement process findings from which it was identified.

The articulation and mapping of the underpinning capacities, combined with the participants’ increased participatory expectations, made it clear that each of the overarching enablers could be characterised as the ‘voices’ that need to be brought together across the transformation processes:

- *The voice of intent: Co-evolutionary design and navigation processes*. Adaptive navigation towards a shared intent, through collaborative visioning, goals-setting, pathways, co-design and planning at all levels, progressively adjusted based on monitoring of emergent urban outcomes, and insights from place-based innovation, experimentation and learning.
- *The voice of experience, behaviours and values: Engagement between decision-makers, stakeholders and communities.* The need for contextualised engagement processes, sometimes assisted by intermediaries, and for multiple purposes including listening to others’ experiences, understanding current behaviours, and ultimately understanding and negotiating diverse values to shape decision-making.
- *The voice of decision-making: Aligned institutions and governance processes.* Decision-makers exercise formal or informal authority that drives outcomes. Institutional innovation and (re)design will be necessary if urban governance at all levels is to move beyond silos and special interests, rebalance power relations, and embrace inclusive, strategic, coherent, evidence-based and transparent decision-making.
- *The voice of expertise: Knowledge co-production, usage and learning processes.* Co-production of increasingly systems-based research agendas and cumulative knowledge, drawing on diverse sources, including all stakeholders and communities as knowledge providers. Usage and learning are supported by open-access knowledge platforms and enhanced people and organisational capacities, that facilitate collaborative sharing, translation, brokering and uptake of new and existing knowledge, and make space for collaborative reflection and learning.

*In this framework it is crucial to note that, in terms of agency, any one actor (whether citizens, communities, stakeholders, researchers, or decision-makers in government, private, professional or NGO sectors) may contribute to all four voices depending on the issue, the stage of the process, and their roles and interests in that context*. It is especially important that all with an interest be involved in the ‘voice of intent’. Working collaboratively towards shared intent, even if never entirely achievable (Kaika 2017; Hulme 2020), is the most significant lever in sustainability transformations (Abson et al. 2017).

#### Comparison with other studies on transformation enablers and capacities

The detail of the EUST framework was informed and tested by comparison with international literature on capacities needed for sustainability and urban transformations. The comparison included the three sources on transformation enablers drawn on in the Webb et al. (2018) urban transformation framing article (i.e. Grimm et al. 2000; Beddoe et al. 2009; Gorddard et al. 2016), and also Wolfram (2016), Waddell (2016), Abson et al. (2017), Scoones et al. (2019), Wolfram et al. (2019), Moser et al. (2019), Kangas et al. 2019, Iwaniec et al. (2019), Hölscher et al. (2019), Shahani et al. (2021) and Grainger-Brown et al. (2022).

The detail of the comparison between each of these sources and the EUST framework is at Additional file 3. In summary it found that the transformational capacities (or equivalent) identified in each study could be quite readily mapped to the four enablers, and to one or more of the capacities in the EUST framework, even though several are defined slightly differently. Each study comes from a somewhat different perspective, and so has its own validity. However, all thirteen have a sustainable development transformation perspective, six in an urban context. Three come through specific capacity windows (Kangas et al. (2019) leadership for change; Iwaniec et al. (2019) transdisciplinary research; Hölscher et al. (2019) governance), but indicate how a broader range of capacities is necessary. The fact that all thirteen can be mapped to a common framework, notwithstanding different starting points, evidences robustness and opportunity for convergence.

Of the other studies Wolfram (2016) is the most explicit in its development of an urban transformation capacities framework. Table 2 therefore also indicates the closest corresponding ten capacities (C1-C10) from Wolfram (2016), and in some cases deliberately uses (with acknowledgement) identical or similar capacity descriptors. In this way the EUST framework builds on, but keeps a direct line-of-sight to that earlier framework which has already been used in a number of case studies.

In summary the EUST framework introduces the concept of the four overarching enablers and the transformation process ‘voices’ they represent and, with the benefit of being able to build cumulatively on the other studies as well as being grounded in the FEA process findings, introduces some additional capacities, and changes in emphasis and descriptors. This indicates that it is possible to aspire to convergent and cumulative frameworks for broader application in urban transdisciplinary processes.

#### KUST framework: Knowledge for Urban Systems Transformation

**Fig. 3:**
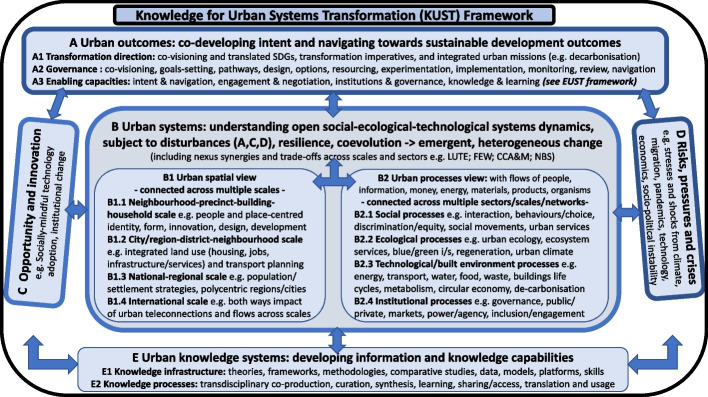
Knowledge for Urban Systems Transformation (KUST) Framework. The transdisciplinary process participants focused on themes that could directly influence future outcomes (via collaborative direction-setting, governance/decision-making and enabling processes: Theme A); supported by knowledge to overcome static views of urban systems, and policy, practice and research siloes (via broader, interconnected and dynamic urban systems view: Themes B, C, D), and access to enhanced knowledge system capabilities (Theme E). Those involved in Theme A processes need to recognise they are themselves also part of the urban systems subject to change (e.g. in Social and Institutional process sub-themes in Theme B2). (See also “Systems-wide frameworks as boundary objects” section in Discusssion  for KUST connection to other recent studies)

A range of research and knowledge themes were identified and mapped throughout the FEA engagement process (Webb and O’Donnell 2019: p.8). This mapping has been used as a transdisciplinary-derived base for the Knowledge for Urban Systems Transformation (KUST) framework at Fig. 3, with the detail enhanced by insights from the EUST framework and some compatible SETS-based descriptors from Zhou et al. (2021) (see also “Systems-wide frameworks as boundary objects” section in Discussion). The KUST and EUST frameworks are complementary. The KUST framework supports EUST ‘Urban systems awareness, knowledge and cumulative understanding’ (Table 2: Capacity 4.3), and the EUST framework supports KUST ‘Enabling capacities’ (Fig. 3: Theme A3).

A future issue-framing and related research agenda would draw together several of the KUST themes to address systems-based urban challenges and missions. As an example, decarbonisation was often raised in the FEA process as a challenge requiring such systems-based changes and knowledge. Within this, a ‘transformational mission’ very relevant to Australia relates to the four interconnected issues in Table 3, on the urban supply, storage and use of renewable energy. It shows how transformational resolution requires an understanding of their interdependencies, drawing on themes across the KUST framework.

**Table 3:** Urban systems and nexus issues: A decarbonisation example

Systems understanding has practical implications for many urban challenges and missions. For example, transformational resolution of the four urban renewable energy issues below needs to appreciate their interdependencies, drawing on each of the knowledge themes in the KUST framework (Fig. 3). Transformation requires capacity-building including shared intent; broader and more strategic urban planning processes with evaluation of options’ co-benefits, trade-offs, and outcomes beyond decarbonisation; multiple demonstrations in cities; supportive policies at state and federal levels; and serious stakeholder and community engagement (*Theme A in* Fig. 3)*.* Renewable options exhibit significant spatial differentiation across five ‘urban fabrics’ (central city walking, inner city transit, outer suburb automobile, peri-urban and rural bioregional, and remote settlement (Seto et al. 2021)), combined with urban process nexus interdependencies *(Theme B*). Options also reflect technology opportunities (*Theme C)* and resilience needs (*Theme D*). Solutions need support from ‘urban systems’ knowledge, and infrastructure for knowledge sharing *(Theme E).* With its high renewable energy potential and take-up, such issues are already significant for Australia. **1. Renewables and local storage opportunities in urban fabrics** ***Issue***: Distributed solar storage options to help stabilise supply to the city-wide and regional grid include individual and community batteries and, in the rapidly emerging future, batteries in electric vehicles; smart technologies that are able to quickly turn appliances on or off; households and businesses with appliances that only turn on when solar is maxing out and have a tariff to reflect this; phase-change material attached to air conditioning that enables excess solar to be stored for later air conditioning; large hot water storage for use later; and even mini pumped-hydro storage in a back yard tank for multiple other local urban functions***. Urban planning responses***: Such transformational options vary with the part of the city and hence different urban fabrics could be enabled to have different storage functions. This avoids recourse to curtailing distributed solar, or else traditional large centralised grid solutions like pumped hydro which are costly and take years to build. ***Co-benefits***: Greater decarbonisation, energy savings and supply resilience. (See further in Green and Newman 2022; Newman 2020a). **2. Electric vehicle automobile dependence and wastage of renewable power** ***Issue***: Switching from diesel or gasoline cars to battery electric vehicles is likely to happen quite quickly as capital costs become equivalent from around 2023, while fuel costs and maintenance of EV’s will be lower. EVs can also contribute power to the grid. However, EV popularity could encourage even greater automobile dependence, which is associated with multiple sustainability issues. It would also waste solar and wind renewable energy that is desperately needed for replacing all fossil fuels including those used in industry for processing materials and making all kinds of products. ***Urban planning responses:*** Urban planning needs to focus on reducing automobile dependence as well as decarbonising all sources of power. This includes rebuilding the city with much greater e-transit, e-rideables and walkability around corridors, precincts and buildings that are net zero, based on solar PV’s, with solutions including closer integration of land use (housing type and densities, jobs, services, public space, biophilic design) and transport, tailored to different urban fabrics. ***Co-benefits***: Greater decarbonisation; reduced congestion and travel times; improved liveability, productivity, and health outcomes. (See further in Seto et al. 2021; Newman et al. 2021). **3. Hydrogen-based wastage of renewable power** ***Issue***: There is growing awareness that renewable energy based (green) hydrogen has major strategic value as a fuel for industrial processing of primary products due to its value as a reducing agent as well as a strong heat source; and for aviation, shipping and some long-distance trucking functions. Most other potential functions for hydrogen in buildings and transport, including in cities, can be better done by solar-based electricity as this is much cheaper than using the same power to make hydrogen, store it and transport it – each step involving significant thermodynamic losses. If hydrogen-based renewable power is being wasted then such practices are also reducing the ability of the world to rapidly decarbonise. ***Urban planning responses***: Incorporating tools such as life cycle carbon and cost accounting assessments alongside spatial planning should evidence the above, and help spatially prioritise proximity of hydrogen production, industrial processing and nearby regional ports, to be transformed into regional hydrogen settlements and economies. ***Co-benefits***: Renewable energy use optimised; new industry and economic growth in regional areas. (See further in Whitehead et al. 2022). **4. Biophilic urbanism and local renewable power** ***Issue:*** Biophilic urbanism has given new life to the planning of cities using natural processes and ecosystems built into and onto buildings and infrastructure. These systems are a major contributor to achieving SDG’s and enabling cooling in a warmer world. However, biophilics can be used to dominate roof spaces and street spaces so that solar energy potential is reduced. This is a nexus between two beneficial uses of urban space and needs to be worked out in every new development and every regeneration project. ***Urban planning responses:*** Analysis will provide multiple options: to plant in streets and use any associated buildings and spaces such as car parks for solar PV; to do biophilic planting and solar provision in spaces not directly on the site of the development but which can be certified as offsets on nearby land, and used by people living or working in the development; and/or by intricate design work that enables both biophilics and solar PV to be integrated into any spaces. Solutions will depend on the kind of urban fabric and many factors such as climate, to enable a systems-based solution. ***Co-benefits***: Balancing biophilic with solar energy solutions provides multiple ecosystem services, health and wellbeing benefits alongside decarbonisation. (See further in Beatley 2017; McDonald et al. 2018).	

### Linking the frameworks to strategies

The final part of the analysis was the mapping of the strategies and actions in the National Strategy (Table 1: Cols. 1, 2) to the individual EUST framework capacities that they have the potential to develop (Table 1: Col. 3). This evidences that collectively the proposed strategies and actions address all of the capacities identified, which is not surprising (but reassuring!) as they drew on the same set of FEA process findings. However, the analysis provides further insights. First, the strategies do not address all the identified aspects of each capacity (see detail in Additional file 2). They focus on those considered most significant and tractable in the current Australian context. Second, Table 1 shows that each of the proposed actions can contribute to multiple capacities. This is particularly evident for Strategy S1.1 (preparing a collaborative and visionary National Urban Policy (NUP)) and Strategy S2.2 (developing a national network of local Knowledge and Innovation (K&I) Hubs). These can therefore be seen as ‘cornerstone strategies’, especially as they also represent the national ‘top-down’ and local ‘bottom-up’ perspectives. Third, delivery of each strategy can be helped by other capacities to the extent they are in place (e.g. Strategic Actions S1.1, S1.2 on developing urban policy and plans, and embedding the SDGs, would be materially assisted by developing several of the knowledge capacities). This demonstrates the high level of interdependency and mutual reinforcement between the capacities (and therefore also the four enablers). This should inform the detailed design and implementation of each strategy so that they develop synergies, and the strategies should be seen as a coherent package to enable urban systems transformation.

**Fig. 4:**
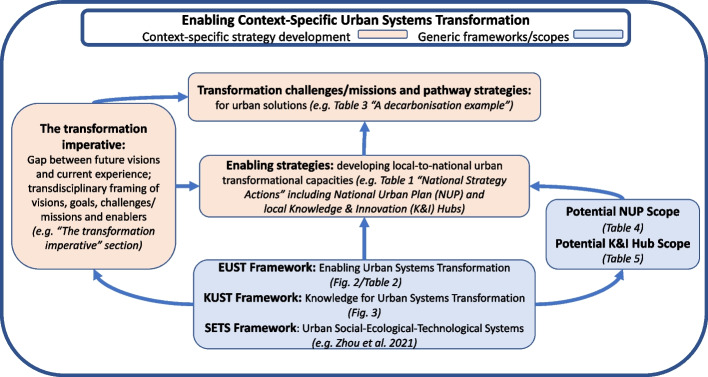
Frameworks enabling urban systems transformation strategies. The EUST and KUST frameworks (and NUP and K&I Hub scopes) can be used as generic boundary objects to facilitate transdisciplinary strategy development in a specific context (e.g. developing national, sub-national, or local urban strategies and missions). These frameworks are also compatible with and complementary to broader SETS-related frameworks. (The examples in parentheses in the three context-specific boxes refer to the equivalent national Australian findings in the text)

Finally, the complementary EUST and KUST frameworks can be boundary objects to facilitate other context-specific transdisciplinary strategy development. The context could be development of a national or sub-national/local urban strategy. This is represented in Fig. 4 where the two frameworks support development of ‘The transformation imperative’ (including framing challenges/missions and enablers) and related ‘Enabling strategies’. This is equivalent to the Australian example in “The transformation imperative” section, and could then support context-specific urban transformation pathways and solutions development. The potential of such boundary objects is discussed further in the “Systems-wide frameworks as boundary objects” Discusssion section.

## Discussion

The first section reflects on the National Strategy co-development process, providing several insights on transdisciplinary approaches to developing urban systems transformation strategies. This is followed by sections that discuss the urban transformation frameworks identified and their potential broader application.

### Reflections on the co-development process

#### Diversity of engagement participants and processes supports whole-of urban-systems and local-national perspectives

The FEA process was designed in part from the experience of the precursor framing study (Webb et al. 2018), and is consistent with general criteria for effective transformation research in being normative, systemic, solution-oriented, challenging the status-quo, and socially robust (Hölscher et al. 2021). It brought together a diverse range of urban stakeholders across sectors and spatial/jurisdictional scales with researchers from multiple disciplines. Distinctive contributions also came from different engagement processes. The city-region workshops enabled a diverse group to engage in collective thinking on visions, issues identification and potential initiatives. They also contributed local perspectives on governance, engagement, and knowledge access and use. The interviews were mostly with national bodies, and were especially helpful on institutional and governance issues across levels and sectors, urban change priorities, and future knowledge and capacity development. The urban researchers had inputs across the spectrum of issues and scales, as most had significant experience in collaborative projects with diverse stakeholders and issues. They were especially aware of significant knowledge gaps, and provided research and systems insights to the broader group of participants. Finally, the Reference Group and roundtables provided inputs on the overall process and outcomes. The combination of diverse participants and engagement processes generated whole-of-urban-systems issues, visions and strategy development, relevant from local-to-national scales.

#### Reflexive social learning on enablers an entry-point to consensus-building...

The FEA process can be seen as an exercise in social learning and reflexivity (Mascarenhas et al. 2021). Diverse participants brought their experiences and knowledge to the process and were expected to be respectful and open to new perspectives from others. Consistent with Castan Broto et al. (2019), a reflexive process proved a good entry point to identify and address a full range of capacities and enablers. The literature warns about the problems of preemptive attempts to find consensus around contentious policy issues (Kaika 2017) and how social difference and uneven power in policy formulation processes disadvantages already marginalised groups (Swynegdouw and Kaika 2014; Brisbois et al. 2019). In the FEA process there was indeed a diversity of views on issues such as the *relative priorities* of urban sustainability goals, and *appropriate pathways* to be pursued, and such contestation is likely when specific sector or local solutions are being developed. However, a remarkable degree of consensus emerged on the overall transformation imperative, enabling capacities needed, and the National Strategy. This suggests that to negotiate contested urban issues, a focus on developing the transformation enablers and capacities required, through an open and reflexive process, might be an entry point to build common ground.

#### … but there are significant challenges ahead

While the consensus was encouraging, the process also identified two significant engagement and commitment challenges. First, the FEA process, while inclusive across institutions, societal sectors and scales, inevitably involved a degree of participant self-selection, attracting people who were well informed and interested (a good thing) but not necessarily representative of the ‘general public’. Being representative is important to understand the full range of community values ascribed to various future priorities, pathways and outcomes. The pilot survey (Costanza-van den Belt et al. 2021) was a first step in establishing the views of citizens not directly involved in urban development issues, other than through their lived experience. However, this would need a substantial extension to be more broadly representative.

Second, it did not prove possible to have the most powerful federal and state government political leaders involved directly in the co-development process, especially across the range of portfolios necessary for a coherent systems approach. Indeed, there was often no obvious ‘departmental sponsor’ of an integrated urban strategy. Government departmental participants understood the need for more systemic strategies but rarely felt they were in a position to significantly change siloed policies and behaviors, a reticence reinforced by politically sustained reduction of public service policy roles and resourcing in recent decades. Furthermore, federal governments have not, over time, fulfilled a consistent leadership role on urban issues, so policy action in this domain is highly dependent on Ministerial interest and external lobbying.

The covid-19 pandemic, starting just a month after the National Strategy launch, severely constrained research sector resourcing, and also made it very difficult to gain federal and state government attention, even though urban sustainability, resilience and post-covid recovery should be linked (Acuto et al. 2020; Newman 2020b). Notwithstanding this distracted environment, with a National Strategy co-developed, FEA has been able to start federal political engagement on behalf of the participant network. With the May 2022 election of a new federal government promoting more progressive policies on sustainable development issues, it is possible to continue seeking policy opportunities, especially as the ten-year frame of the National Strategy means it remains highly relevant. There is also potential to connect with increasingly progressive state governments and, for certain key National Strategy components such as the proposed Knowledge and Innovation (K&I) Hubs, to build on existing initiatives (e.g. CSIRO Urban Living Labs, various sector-specific research Hubs, and a new iHub network^3^ (Newton and Frantzeskaki 2021)).

### Systems-wide frameworks as boundary objects

The ‘enabling urban systems transformation’ (EUST) framework (Fig. 2, Table 2) has been developed from the FEA process outcomes, and also built in a deliberately cumulative way on other related frameworks, and most explicitly that of Wolfram (2016). The latter has itself been used in case studies and capacity assessments across the global South and North (Wolfram et al. 2019; Castan Broto et al. 2019) and across very different socio-political systems (Meyer et al. 2021; Shahani et al. 2021). Most of these studies have focused on city-region to local scale initiatives, though some draw attention to the need for studies across geographic scales (Castan Broto et al. 2019; Borgstrom 2019), and refer to the influence of national policies (Wolfram 2019).

However, the EUST framework has at its foundation a ‘national’ approach seeking to support coherent action by multiple actors from local to national scales and across sectors. Many issues raised required a common (or at least coordinated) response at higher national and sub-national levels, and synergies were evident across levels. The literature also indicates that, to influence complex sustainable development outcomes, it is crucial to include the reciprocal interactions of institutional structures and stakeholder agency across local to international scales (Riechers et al. 2022), and the particular significance of the national or ‘meso’ scale institutions (Fischer and Newig 2016; Loorbach and Shiroyama 2016; Ehnert et al. 2018). The EUST framework thus adds several new perspectives to a cumulative and robust body of urban knowledge, providing a boundary object to assist transdisciplinary processes across urban contexts and local-to-national scales. It may also inform sustainability transformations more generally as the urban context is among the most complex of systems.

The Knowledge for Urban Systems Transformation (KUST) framework also arose from the FEA process and provides a window into the dynamic urban systems from the perspective of those developing change strategies, as it enters via transformation imperatives, directions-setting, governance and enablers (Fig. 3: Theme A). Appreciating that many synergies and trade-offs in urban systems are currently ignored, it identifies the range of urban systems themes (Fig. 3: Themes B,C,D) that might be brought together to better frame urban challenges, missions and knowledge (e.g. Table 3). Some problems of isolated research and innovation ‘projectification’ can also be addressed through more broadly framed programs (Nylén 2021).

Supporting this systems approach is current research on nexus issues across subsets of urban systems (as referred to in the KUST framework and also relevant to many of the synergies and trade-offs in Table 3):

- integrated urban land use, transport and environment (LUTE) (Acheampong and Silva 2015);
- urban and hinterland nexus between food-energy-water (FEW) (Covarrubias 2018; Zhang et al. 2019) and extension to include waste (FEWW) (Valencia et al. 2022);
- multiple cross-sector benefits from nature-based solutions (NBS), blue/green infrastructure (BGI) and urban and hinterland ecosystem services (Haberman and Bennett 2019; Filho et al. 2020; Newton and Rogers 2020);
- interactions between urban climate change adaptation and mitigation (CCA&M) strategies (Urge-Vorsatz et al. 2018);
- linking urban metabolism, circularity and digitalisation (D’Amico et al. 2022); and
- linking urban systems, sustainability and public health (Taylor and Howden-Chapman 2021).

There are in fact many valid ways of representing transformational urban missions (e.g. NASEM 2016; Mazzucato 2018; JPIUE 2019) and research/knowledge themes (Wolfram and Frantzeskaki 2016; Wolfram et al. 2017; Ramaswami et al. 2018; Prieur-Richard et al. 2019; Frantzeskaki et al. 2021; Hölscher and Frantzeskaki 2021; Zhou et al. 2021). While variety in the research/knowledge themes is unsurprising given the intrinsic complexity and diversity of urban issues, all the above studies are based on a complex-systems view of ‘the urban’, and several call for a more convergent ‘urban science’. Indeed, Additional file 4 shows how it is possible to map the research/knowledge themes proposed in these studies to those in the KUST framework, indicating opportunity for further convergence.

Zhou et al. (2021) explicitly address convergence, based on urban SETS as complex, open and heterogeneous systems, and synthesise a nested set of conceptual frameworks to ‘operationalize the theory in actual situations’. The KUST and EUST frameworks are aligned to this ambition and its urban systems foundation, providing complementary boundary objects to help drive such operationalisation. Having been developed inductively from an extensive transdisciplinary process, and then connected to recent literature, they are consistent with the call of Schlüter et al. (2022) to advance sustainability theories and ‘change making’ by bridging reflexive and transdisciplinary practice insights back to concepts and theory. They are positioned from the perspective of those developing transformational strategies, using language derived primarily from a practical transdisciplinary process, with a level of detail sufficient to facilitate engagement between diverse stakeholders, without being overwhelming or too context-specific. They can assist urban transformation strategies through transdisciplinary processes in other contexts (Fig. 4), including other countries, and from local to city-region to national levels; and also contribute a small step towards a more convergent, cumulative and transdisciplinary ‘urban science’.

### Some high leverage enabling strategies

#### The cornerstone strategies may have broader application…

As noted in the “Linking the frameworks to strategies” Results section the National Urban Policy (NUP) and the network of Knowledge and Information (K&I) Hubs can be seen as ‘cornerstone’ proposals in the National Strategy. Equivalent strategies are relevant in other national contexts. Thus, the New Urban Agenda proposes that all countries develop National Urban Policies (UN-Habitat 2016) and the K&I Hubs are consistent with the growing interest in Urban Living Labs (ULLs) or equivalent (Steen and van Bueren 2017; Chroneer et al. 2019; Hossain et al. 2019) and urban observatories/knowledge exchanges (Dickey et al. 2022).

To support such proposals, Tables 4 and 5 provide potential scopes of a NUP and of K&I Hubs, that encompass the development of all capacities in accordance with those identified for the corresponding NUP and Hub strategies in Table 1. Table 4 is also consistent with international guidance on national urban policies, which to-date is only partly met in practice (UN-Habitat and OECD 2018; OECD, UN-Habitat and UNOPS 2021). However, drawing on Table 1, it extends this guidance in several areas of ‘capacity building’. For K&I Hubs Table 5 includes the facilitation of local innovation and experimentation across social, ecological and technological domains (van der Jagt et al. 2020; McPhearson et al. 2022), embracing the plurality of local experiential knowledge (Miller et al. 2011; Nevens et al. 2013; Grabowski et al. 2019); but also aims to significantly enhance access to relevant learning and knowledge (locally, and nationally through a network of hubs), with an emphasis on ‘systems’ perspectives to complement any more specialised ‘hubs’. The Table 5 scope therefore includes, but is significantly broader than many ULLs (typically local innovation/ experimentation-oriented) and observatories (often more data-driven), with additional focus on national learning, knowledge sharing and scaling for greater transformational impact (Evans et al. 2021; Miller et al. 2021). While the broader scopes in Tables 4 and 5 will not always be feasible initially, they provide (as shown in Fig. 4) an input for those designing enabling strategies to evaluate which elements are most immediately relevant in their context. Being linked by the urban capacities that each is aiming to develop, they also make it easier to ensure that top-down and bottom-up strategies are mutually supportive.

**Table 4:** National Urban Policy (NUP) scope and capacities supported

Potential scope of a National Urban Policy ***[and Capacities supported]***	
**(1) Urban visioning and navigation for national development and international commitments** ***[Capacities 1.1-1.3, 3.3]*** • Collaborative national urban visioning and goals-setting, aligned with translated SDGs, international commitments (UNSDGs, NUA, Paris Agreement, CBD, Sendai), and emerging strategic trends, challenges, risks, opportunities, responses. • National urban performance indicators, and monitoring and navigation processes, incorporating insights from upscaling of local urban research and innovations • Guidance for line-of-sight equivalent at sub-national scales, including strategic urban planning processes, design and governance principles	
**(2) Horizontal and vertical policy coordination and coherence** ***[Capacities 1.3, 3.1-3.3]*** • Develop co-ordination and coherence, horizontally across social, economic, environmental, innovation, resilience, spatial policy areas at national level; and vertically between levels of government, clarifying roles, responsibilities and resourcing principles. Includes national settlement strategy with system-of-cities, and urban-rural connections. • Develop implementation mechanisms with legal, regulatory, planning and financial tools; and accountability and integrity processes	
**(3) Stakeholder engagement and participation:** ***[Capacities 2.1-2.2]*** • Promote engagement and participation of governments/regulators with all stakeholders/ communities; develop engagement best practices platform; demonstrate engagement by inclusive approach to NUP development	
**(4) Financial and resource capacity building:** ***[Capacities 3.1-3.5]*** • Develop financial and resourcing principles and access across levels, including innovative sources • Influence urban directions through national funding for collaborative urban infrastructure and place-based programs	
**(5) Policy-practitioner-research and knowledge/innovation capacity building:** ***[Capacities 1.3, 2.3, 3.4-3.6, 4.1-4.5]*** • Contribute to policy–practitioner–researcher capacity-building programs and collaborations. • Develop national urban systems research and innovation programs with challenge/ mission-oriented priorities and guidance for funding; and national urban data, knowledge and innovation sharing platforms, with space for collective reflection and learning. Ensure robust urban-scale data and indicators to support governance and navigation. • Contribute to national network of distributed ‘hubs’ as catalysts for co-development and sharing of local knowledge, innovation, learning, engagement and capacity-building ***(see*** Table 5***for potential Knowledge & Innovation Hub scope)***	

Potential scope is a synthesis based on the range of urban capacities a NUP can support (see Table 1: Col. 3 at *National Strategy S1.1*); OECD, UN-Habitat and UNOPS (2021) (Global State of National Urban Policy); and OECD (2019) (Principles on Urban Policy). Mapping is shown to capacities in the EUST framework (Table 2)

**Table 5:** Knowledge and Innovation (K&I) Hubs scope and capacities supported

Potential scope of Knowledge and Innovation Hubs ***[and Capacities supported]***	
**(1) National and international networking:** ***[Capacities 2.2, 3.3, 3.5, 4.1, 4.3]*** • As the local hub of a national K&I network and platform, contribute to national and international sharing of capabilities, knowledge and solutions; de-contextualised innovation acceleration, up-scaling and out-scaling; ‘whole-of-urban-systems’ understanding; toolkits/boundary objects to support strategic urban planning and community-centred engagement; and knowledge input to a National Urban Policy ***(see*** Table 4***for potential National Urban Policy scope)***	
**(2) Local cross-systems innovation collaborations**: ***[Capacities 1.3, 2.1, 3.1-3.2, 3.4, 4.1-4.3]*** • Facilitate transdisciplinary issue framing, experimentation, incubation, business models, and real-life solutions, combining diverse local engagement and experiential knowledge with national/global knowledge and practice, into social, ecological, technological and governance innovations • Provide space and technologies for visualisation and open discussion of possible urban futures, challenges and proposals, with citizens, public and private actors, researchers	
**(3) Local cross-systems knowledge development, usage and learning:** ***[Capacities 3.5, 4.1-4.5]*** • Work with local/regional stakeholders, citizens and other more issues-based research/knowledge providers, to co-develop understanding of priority urban missions and related systems interdependencies and dynamics; identify priority knowledge needs and sources, including long-term data-gathering for research/modelling and KPI monitoring; and facilitate optimum use of *existing* knowledge and data, and transdisciplinary co-production of *new* knowledge • Provide knowledge brokering, curation, analysis/synthesis, translation, presentation services; support reflexive social and organisational learning, collaborative research-policy-practice knowledge capabilities, and mainstreaming of knowledge into practice	
**(4) Supporting local strategic urban planning, governance and leadership:** ***[Capacities 1.1-1.2, 3.1-3.4, 3.6]*** • Support urban planning/coordinating agencies in developing their central roles of facilitating inclusive and coherent collaborative visioning, goal-setting, policy, planning, investment, innovation, monitoring and navigation, at metro/region to local scales • Support local formal and informal leadership and decision-making, including empowerment and leadership of local communities and stakeholders	
**(5) Local relationships and long-term trust-building**: ***[Capacities 2.1-2.3, 4.1-4.2, 4.5]*** • Develop cross-sector/scale and cross-disciplinary trust through networks and collaborations, including as an independent adviser, intermediary and facilitator; providing a safe space for collective learning and negotiation of conflict; and open knowledge-sharing and collaboration with other knowledge/research providers and hubs	

Potential scope is based on the FEA process outcomes that led to the National Strategy proposal for a network of K&I Hubs, and the range of urban capacities potentially supported (see Table 1: Col. 3 at ***National Strategy S2.2*)**. Mapping is shown to capacities in the EUST framework (Table 2)

#### …but strategies will also depend significantly on structural and systemic contexts…

The translation from the above frameworks and strategy scopes into context-specific strategies will depend substantially on local socio-economic-political starting points, and the nature and extent of structural and systemic challenges (Scoones et al. 2019). Translation for Global South countries is especially important. It is projected that 90% of global urban population growth of 2.5b by 2050 will be in Asia and Africa, which would then have 74% of the total global urban population (UNDESA 2019). Global South cities have more extreme challenges than those where most urban research is carried out (Nagendra et al. 2018; Bai et al. 2018; Mahendra et al. 2021). These include large informal settlements and economies, extreme poverty, poor access to basic services, limited finance and resources, and unique institutional characteristics arising from blends of colonial, discriminatory and either authoritarian or fragile origins.

In developed countries, challenges are less intense, but include societal discrimination, neoliberal approaches and capitalist excesses, leading to recognitional, distributional and procedural inequities (Leach et al. 2018; Mazzucato 2021), and the need for new government responses to radical social, environmental and technological change (Geels et al. 2021). Similar issues were raised in the Australian context, including impacts of neoliberal government policies, private sector developer influence, social inequities and marginalisation, and lack of policy coherence across sectors, agencies and levels. These are just examples of the structural and systemic contexts, often reflecting institutional barriers to change and power imbalances (Flyvbjerg 2004), that will suggest necessary transformation strategies.

#### … and the enablers framework can help focus on related power imbalances

In the EUST framework the ‘*voice of intent’*, requiring co-evolutionary design and navigation (Kallis and Norgaard 2010) and in many cases fundamental shifts in prevailing beliefs, values, worldviews and paradigms (Abson et al. 2017; O’Brien 2021a, 2021b), is central to establishing a normative direction for sustainable development, and then maintaining movement towards what is likely to be a constantly moving target (Castan Broto et al. 2019). It depends on input from, but should then also inform, the other three enablers. To be in the interests of citizens, intent would be primarily driven by the ‘*voice of experience, behaviour and values’*, further informed by the best available knowledge (the ‘*voice of expertise*’) and institutional advice (the *‘voice of decision-making*’). Institutions would then adjust to deliver on agreed intent in the collective interest. This view reflects a relational or mosaic (rather than entity or hierarchical) view of governance (Buijs et al. 2019).

The reality is usually very different to this. Current decision-making institutions exercise great power and often entrench current practices (Markolf et al. 2018). They control not only the decision-making processes, but also the way the other three ‘voices’ are developed and heard, and are often the most significant barrier to transformational change (Abson et al. 2017; Ernstson 2021; Patterson 2021). Intent becomes distorted by institutional interests and power. Engagement by decision-makers is often token, and the influence as to which knowledge is commissioned or used is highly politicised, with local experiential knowledge marginalised (Rozance et al. 2019).

The EUST framework helps focus on how structural and systemic power relations may need to change to ensure urban outcomes serve citizens’ interests, by thinking of the four enablers as ‘voices’ contributing to the transformation process (Fig. 2), with multiple actors contributing to each but currently with very different weightings. The framework then also recognises the need to address systemic and structural institutional change at the capacity level, especially through Enabler 3 where individual capacity characteristics (Table 2 and Additional file 2) include developing *systemic* institutional alignment and coherence, *structural* innovation and redesign of institutions, *empowerment* of stakeholders and communities, and *formal and informal leadership* articulating new narratives, bridging barriers, and motivating engagement and collaboration. The other three enablers include *inclusive processes of intent formulation*, *genuine engagement* with those impacted, and *co-production of knowledge*, each also helping drive institutional change. In a particular situation these are likely to require collaborative, systemic, goal-setting and evolutionary strategies (Grabowski et al. 2017; Monstadt et al. 2022) that combine the impact of entrepreneurs, communities, activists and collaborations supported by courageous change leaders from within current institutions (Waddell 2018), and recognition that institutional and societal change is influenced by developments and actors beyond the local scale (Geels 2011; Sareen and Waagsaether 2022).

The vesting of authority to institutional power may well be an inevitable by-product of the development of civilisation, with cities its ‘greatest invention’ (Glaeser 2012). However, where current power relations detract from sustainable development, strategies to enable transformation through the lens of the four enabling ‘voices’ and their underpinning capacities can be a critical opportunity to address the ‘imbalance of the voices’.

## Conclusions

There is growing awareness of the need for transformative urban development, and that a systems-based approach can help. An extensive transdisciplinary process has co-developed a National Strategy to ‘enable urban systems transformation’ in Australia from local-to-national scales. The approach evidenced how diversity in participants and engagement processes can develop whole-of-urban-systems- and local-to-national perspectives, and that reflexive social learning can help build consensus. However, it also evidenced the importance of stronger and more consistent leadership, especially at federal and state government levels where greater powers exist to facilitate transformation across sectors and scales.

Analysis of the outcomes also demonstrated that while urban transformation *strategies and solutions* will be context-specific, there are underlying *frameworks and strategy scopes* that are more generic. The *Knowledge for Urban Systems Transformation (KUST)* framework built on the transdisciplinary process outcomes, and can help frame urban challenges, missions and knowledge programs. The complementary *Enabling Urban Systems Transformation (EUST) framework* also built on the transdisciplinary outcomes, and cumulatively on transformation capacity frameworks developed by others. It identifies four overarching enablers or ‘voices’ in the transformation process, and the underpinning capacities to be considered in an enabling strategy. It also provides a focus on structural and systemic power imbalances that need to be addressed to ensure there is stakeholder and community inclusion in each of the four enabling processes. Finally, the potential scopes of a National Urban Policy and a network of local Knowledge and Innovation Hubs are extended, and made mutually supportive as top-down and bottom-up ‘cornerstone strategies’, by identifying the full range of urban capacities that each can support.

These frameworks and scopes have potential, as transdisciplinary boundary objects, to assist issue framing and strategy development in other countries (Fig. 4). They are also consistent with calls for a more convergent, cumulative and transdisciplinary ‘urban science’, and their further development and practical deployment should help confirm what is similar across urban contexts, and broaden perspectives on the many context-specific and contested issues.

It is recognised that this is a continuing journey and there will be valid alternative and complementary ways to enter the complexity of urban settlements and cities. Nevertheless, progress on the related ambitions of *cumulative knowledge building, enabling urban systems transformations* and *rebalancing power relations*, is critical if communities, stakeholders and decision-makers are to navigate with some speed towards more sustainable urban development.

## Supplementary Information

**Additional file 1.** Summary of current issues and 2030-50 visions based on the nine city workshop visioning exercises.

**Additional file 2.** Enabling Urban Systems Transformation (EUST) framework: elaborating on the underpinning capacities.

**Additional file 3.** Mapping of other published ‘sustainability’ and ‘urban’ systems transformation capacity frameworks to the EUST framework enablers and capacities (EUST framework at Fig. 2 and Table 2 in main article text).

**Additional file 4.** Mapping of key themes in urban systems ‘science/knowledge/research’ articles to the KUST framework themes (KUST framework at Fig. 3 in main article text).

## Data Availability

Detailed supporting information is in the supplementary materials (Additional files: Af1 on urban issues and visions; Af2 on details of urban transformation capacities; Af3 on mapping other literature with EUST framework urban systems transformation enablers and underpinning capacities; Af4 on mapping other literature with KUST framework urban systems knowledge themes).

## Abbreviations

AUST: Australian Urban Systems Transformation
BGI: Blue-green infrastructure
CBD: Convention on Biological Diversity
CCA&M: Climate Change Adaptation and Mitigation
EUST: Enabling Urban Systems Transformation
EV: Electric Vehicles
FEA: Future Earth Australia
FEW: Food-energy-water
FEWW: Food-energy-water-waste
KUST: Knowledge for Urban Systems Transformation
K&I: Knowledge and Innovation
LUTE: Land Use Transport and Environment
NBS: Nature-based Solutions
NUA: New Urban Agenda
PV: Photo-voltaic
SDG: Sustainable Development Goals
SETS: Social-ecological-technological systems
UNCBD: UN Convention on Biological Diversity
UNDRR: UN Disaster Risk Reduction
UNFCCC: UN Framework Convention on Climate Change

## Footnotes

1 Examples raised were new spatial data sources; the Internet of Things; real-time sensor data streams; infrastructure process flows management; strategic and real-time operational use of machine learning and artificial intelligence; digital twins/Building and Precinct Information Modelling/assets engineering and management for design-build-operate-maintain; drone and transport technologies; future communications tehnologies (5G, 6G); and interoperability data standards for cross silo analysis.

2 Australian-based urban research sources identified included Cooperative Research Centres for Reliable, Clean, Affordable Energy (RACE) for 2030, Low Carbon Living, Water Sensitive Cities, Spatial Information, Transport and Mobility (iMove), and Construction Innovation; Centre of Research Excellence in Healthy, Liveable and Equitable Communities; the Clean Air and Urban Living and Sustainable Communities and Waste Hubs within the National Environmental Science Program; the Australian Housing and Urban Research Institute; the Sustainable Built Environment National Research Centre; the Australian Urban Research Information Network; CSIRO Urban Living Labs; the Australasian Cities Research Network; and multiple university centres, programs and projects.

3 The iHub is a National Urban Research Platform representing a network of collaborating university urban laboratories located in Australia’s four largest capital cities capable of synchronously engaging in the full spectrum of strategic activities listed in Table 1, with experts, government and industry stakeholders and citizens. It is readily scalable with capacity to focus on local to national projects.
