# Supplementary material for: Enabling urban systems transformations: co-developing national and local strategies
Source: Urban Transform. 2023 Feb 20;5(1):5. doi: 10.1186/s42854-023-00049-9 (PMC9939254; doi:10.1186/s42854-023-00049-9)
Supplement: Supplementary file 1 — Additional file 1. Summary of current issues and 2030-50 visions based on the nine city workshop visioning exercises. [file 42854_2023_49_MOESM1_ESM.docx]

## Additional file 1 Summary of current issues and 2030-50 visions based on the nine city workshop visioning exercises

### Table Af1.1 Summary of current issues and 2030-50 visions based on the nine city workshop visioning exercises

| **Sustainable development outcome area** | **Current issues common across cities** | **Current issues differentiated across cities** | **Typical 2030-50 vision words (*and Tensions*)*** |
| --- | --- | --- | --- |
| Overall images of the future | Lack of sustainability, resilience, transformative capabilities. Declining liveability. | Liveability decline concerns greatest in the largest cities (Sydney, Melbourne); significant intra-metropolitan and city-region variability in levels of amenity and access | transformed, transitioning, emerging, resilient, adaptive BUT ALSO confusing, controversial, chaotic, Mad Max  *Tension: Utopian vs Dystopian?* |
| Environmental and natural resources futures | Natural resources overuse. Excessive waste, pollution, GHG. Land and water supply and quality. Loss of green space. Degraded vegetation. Biodiversity loss. | Specific climate change risks (e.g. heatwaves W Sydney, Darwin, SEQ; bushfires Canberra; water supply and quality issues (e.g. SEQ, W Sydney, Perth, Darwin) | sustainable, intergenerational, within planetary boundaries, regenerative, blue/green/biophilic infrastructure, climate ready, cool, decarbonised - net zero/positive carbon |
| Social, cultural and psychological futures | Social justice, inclusiveness and growing inequity – poverty, access to services, housing. Cultural diversity as challenge and opportunity. Culture and heritage undervalued | Cost of living drivers (e.g. housing in Sydney, Melbourne, Canberra, SEQ; food, fuel in Darwin)  Proportion of indigenous ‘first nations’ peoples (Darwin, Alice Springs)  Nature of local identity | safe/secure; equitable/fair; inclusive; socially connected; tolerant; attuned to diverse cultures; cultural; liveable; healthy; confident; courageous; vibrant; dynamic; relaxed; familiar  *Tension: dynamic, embracing change vs relaxed, familiar?* |
| Economic, growth, employment and technology futures | Managing innovative but disruptive industry, technology and work/jobs change, including for energy, circular economy, smart cities | Extent of vulnerability to economic change and boom-bust cycles (e.g. greater for Adelaide, Perth, Darwin)  Different sectors with economic challenges and opportunities | prosperous; new economy/employment opportunities; innovation; automation; de-growth; beyond GDP; disruption of work  *Tension: growth vs de-growth?*  *Tension: secure vs disrupted employment?* |
| Governance, engagement and decision-making futures | Overcoming siloes. Need to reflect integrated urban systems, trade-offs and synergies. Low trust in institutions. Urban planning process and implementation gaps and distortion. | Different metropolitan structures (e.g. Sydney’s Greater Sydney Commission; Melbourne Councils Partnerships; larger size of Brisbane City Council etc) | Well governed, collaborative, shared value, community voice, empowered, integrated, system-based decisions, evidence-based outcomes, science-based targets |
| Urban form, infrastructure and services futures | Need for national urban settlement strategy. Managing population growth and aging. Excessive urban sprawl but poorly designed densification, need place-based design with public spaces. Need more integrated land use and transport, active transport, congestion reduction. Access to employment, services, affordable housing. | Extent of growth, sprawl and liveability pressures (e.g. greater for Sydney, Melbourne, SE Queensland/ Brisbane)  Proximity of significant regional centres (e.g. less relevant for Adelaide, Perth, Darwin) | Sense of place, compact, connected (socially, physically, virtually, environmentally), with more public spaces and active/ attractive streets, accessible, walkable, localised |

*[More detail of 2030-50 vision words in fourth column is at Table Af 1.2 below]

### Table Af1.2 Synthesis of workshop word clouds/concepts for 2030-50 city visions

This elaborates on Table Af1.1 with a synthesis of all the words/concepts that emerged in the word clouds sessions and subsequent discussions in the nine FEA process workshops. Following the workshops, they have been grouped into the common themes below.

- They give a rich picture of the overall coverage of participant visions and the way they thought about the future for their city. There were many areas of consistency between participants and across cities.
- However, there are also some tensions and different perspectives evident within some of the themes below, which would be useful to explore in future scenario development/analysis.
- It was clear a lot more tensions could emerge in any attempts to prioritise and deliver on these aspirations.
- There were also some differences in emphasis between individual cities (not shown here – see Table Af1.1).

**Synthesis of workshop word clouds/concepts for 2030-50 city visions**

**A. Overall images of the future city**

- Brave-new-world, Transformed, Transition, Emerging, Adaptation, Revitalised
- Different, Revolution, Confusing, Controversial, Chaos, Mad-Max, On Mars, Survival
- Resilient, Adaptive, Responsive, Agile, Versatile, Self-sufficiency

*Tension: Utopian vs Dystopian?*

**B. Environmental and natural resource futures**

- Sustainable, Intergenerational, Carers-of-everything, Holocene-restored, Limits, Within social and planetary boundaries, Regenerative, Net positive
- Environmentally sustainable, Environmental leader, Nature, Ecosystem, Eco-aware, Biophilic, Deep ecology, Permaculture, Green, Green corridors, Tree-lined, Urban forest, Vegetated, Biodiverse, Rewilding, Eco-restoration, Green-buildings
- Climate-ready, Designed-for-climate, Cool, Cooler, Shady
- Climate change, Growing carbon-positive, Zero-carbon, Fuelled renewably, Hydrogen economy, Energy efficient
- Clean, Fresh, Unpolluted, Swimmable
- Circular economies, Waste-free, Zero-waste Water reuse, Sewage, Plastics

**C. Social, cultural, emotional and psychological futures**

*Social and cultural futures*

- Security, Safe, Unafraid, Sheltered, Trust, Low-crime, Refrain from war
- Just, Fair, Responsible, Ethical, Inclusive, Equitable, Egalitarian, Affordable, Famine, Hunger-free, Equitable housing, Frustrated-youth, Proactive Socialism
- Socially-connected, Community, Neighbourhoods, Decentralised, Local, Communal-vs-individual, Personal, Family
- Humane, Caring, Nurturing, Kind, Compassionate, Tolerant, Respectful, Accommodating, Understanding, Valued, Harmony, Mindful
- Acknowledge-history, Indigenous, Treaty, Heritage, Multicultural, Diverse, Socially-diverse
- Cultivated, Cultural, Arts
- Understanding inter-generational drivers: GenX. GenY, Millennials, GenZ

*Emotional and psychological futures*

- Liveable, Healthy, Wellbeing, Healthy-Mind-Body-Spirit, Soul, Beautiful, Aesthetics
- Comfortable, Relaxed, Stress-free, Familiar, More mature, Serene, Slow, Peace
- Confident, Open-minded, Positive, Embracing, Progressive, Embracing change, Enlightened, Courageous, Freedom
- Vibrant, Dynamic, Active, Alive, Energetic, Flourishing, Joyous, Happy, Fun, Convivial

*Tension: dynamic, embracing change vs relaxed, familiar?*

**D. Economic, growth, employment and technology futures**

*Economics, growth and employment futures (some clear tensions evident here)*

- Prosperous, Rich, Enriched, Economic driver, New economy, Networks-not-markets, Services-not-goods, Business opportunities, Added-value, Good supply chains,
- Strong links between jobs and population growth, Employed, Universal-employment, Disruption of work, Secure-employment, Career-of-everything, Skilled, Industrious, Efficient
- Low growth, Zero-growth, De-growth, Reduced-population, abandon-growth-mantra, Growth Beyond GDP

*Technological futures*

- Technology, Innovative, Smart, Digital, Automated, Automation, AI, Cashless

*Tension: growth vs de-growth?*

*Tension: secure vs disrupted employment?*

**E. Governance and decision-making futures**

- New-leaders, Clarity-of-purpose, Well-planned, Well governed, Improvements-in-governance-and-politics
- In tune government, Collaborative, Cooperative, Unified, Bi-partisan, Shared-value, Balance
- Integrated, Joined-up, Cross border links, System-based decisions
- Evidence-based outcomes, Science based targets, Research, Communities-of-practice
- Community-voice, Representational, Empowered, Self-determining

**F. Urban form, infrastructure and services futures**

- Well-infrastructured, Serviced
- Sense of place, Better suburbs, Compact
- Connected (socially, physically, virtually, environmentally), Interconnected, Accessible
- Public transport-rich, Metro-served, Passenger and freight segregation, Car-free, Uncongested
- Active transport, Cycle friendly, Pedestrian-friendly, Walkable

**G. Other images we and others will have of our city in the future**

- International, Links to Asia Pacific region, Linked to other regions, Cosmopolitan, Feels like a big city not a small town, Politically-powerful
- Leader, Frontrunner, Exemplar, Showcase, Famous, Pride, No cultural cringe
- Welcoming, Friendly, Loved, Destination
