# Supplementary material for: Enabling urban systems transformations: co-developing national and local strategies
Source: Urban Transform. 2023 Feb 20;5(1):5. doi: 10.1186/s42854-023-00049-9 (PMC9939254; doi:10.1186/s42854-023-00049-9)
Supplement: Supplementary file 2 — Additional file 2. Enabling Urban Systems Transformation (EUST) framework: elaborating on the underpinning capacities. [file 42854_2023_49_MOESM2_ESM.docx]

## Additional file 2 Enabling Urban Systems Transformation (EUST) framework: elaborating on the underpinning capacities

This file elaborates on the four enablers and their underpinning capacities for urban systems transformation (Table 2 in the main article).

For each capacity there are a few dot-points that characterise and help operationalise the capacity, followed by brief text on how the FEA process findings helped identify each capacity. As shown in Table 1 in the main article, proposals were also generated in the FEA process to respond to the process findings, and therefore to building the capacities identified below. These were distilled in the National Strategy.

### **ENABLER (1) Co-evolutionary design and navigation – ‘The voice of intent’**

#### **CAPACITY 1.1 Co-evolutionary intent, design and navigation**

##### Characteristics

- Adaptive navigation with long-term visioning, planning and delivery informed by step-out innovations and outcomes monitoring
- Flexible transitional pathways and plans respond to emergent systems monitoring and outcomes, while keeping transformational focus
- Longer term visions and goals also change when necessary but less frequently

##### FEA process findings

Participants identified long term urban visioning and planning, step-out innovations, and responding to emergent and sometimes unexpected outcomes, as essential components of expressing intentional design of urban futures, and navigating towards the future; but also noted that governments were not typically good at this given short term political cycles In the more academic language of some of the participants, this was noted as consistent with transformative development being a co-evolutionary (re)design process of complex adaptive urban systems, requiring (even if there is shared initial intent as to how to shape the future) an acceptance of emergent rather than predictable outcomes; and the need for flexible transition pathways and adaptive navigation to steer towards medium and longer-term visions and goals that will themselves inevitably change over time. The transformation was seen as co-evolutionary in at least two senses – between all the elements of the complex and emergent urban social-ecological-technological systems (SETS); and in the necessary interdependencies between long term urban visioning and emergent shorter-term outcomes, including learning from the multiplicity of step-out innovations and experiences.

Getting broader recognition of the centrality of such adaptive navigation to delivering on transformational intent, and then putting it into practice, is perhaps the most fundamental transformational capacity of all – and yet from the findings of the FEA process, probably the hardest and least achieved.

#### **CAPACITY 1.2 Shared visioning, scenarios, goal-setting, pathways, planning and performance**

##### Characteristics

- Participatory, 3-horizon, place-, context- and identity-based visioning, narratives and goal-setting
- Alternative scenarios, flexible pathways, systems leverage points, plans and performance monitoring address multiple interacting risks and opportunities
- Achievable goals and targets recognise trade-offs and synergies between interdependent urban systems

##### FEA process findings

Participants thought that this capacity should be facilitated by government-led strategic urban planning, especially at the metropolitan/regional and local levels. The FEA process found that while metropolitan and local plans exist in Australia for major cities and settlements, there are significant deficiencies. They generally do not reflect scenario-based visioning, translated to flexible pathways, and shared goals based on meaningful negotiation of diverse values. Nor do they reflect the level of adaptive management (including effective urban systems measures and monitoring, and processes for scaling up the learning from innovation, experimentation and experience) to support Capacity 1.3 and the navigation role referred to in Capacity 1.1.

The FEA engagement process itself included broad stakeholder visioning (e.g. Additional file 1), consistent with multiple time horizons, and demonstrated the interest in and potential of developing multi-level (but ultimately place-based) visioning between highly diverse participants. The desire to mainstream this into government planning was seen as needing to be combined with alternative scenario development and collaborative selection from alternative transition pathways; and with goals and targets linked to local translation of the SDGs. There was a range of ideas on performance measures to significantly enhance the utility of the current federal government’s National Cities Performance Framework (NCPF). Participants believed all the above needed to be based on understanding, negotiation and incorporation of diverse stakeholder and community values, and related trade-offs and synergies informed by cross-sector and cross-scale knowledge.

#### **CAPACITY 1.3 Experimentation, innovation, and cessation, recognising the evolutionary phases of introducing the new and ceasing the outdated**

##### Characteristics

- Investment in experiments and innovations (institutional, social, technological and/or ecological)) needed at both local and broader urban scales, using co-design and co-development approaches
- Government, investor, market and citizen decisions progressively remove current regime path-dependency
- Innovation and cessation experience progressively informs and adjusts urban visions, goals and plans

##### FEA process findings

The participants recognised the need for co-designed and co-developed step-out innovations and experiments at the local level, to meet local needs and to demonstrate the broader transformational potential of such innovations including dismantling of traditional path dependencies. This included social and institutional innovation as well as technological innovation. It was clear that innovation is also needed at broader urban scales (e.g. transformational infrastructure potential in areas such as transport, energy, water, communications, social housing, facilitating education/technology precincts and state/federal designated employment centres). The FEA process therefore confirmed the need for both top-down and bottom-up initiatives, and very strongly proposed collaborative innovation hubs be developed at the local level, which could also play a key role in knowledge sharing.

### **ENABLER (2) Engagement between decision-makers, stakeholders and communities – ‘The voice of experience, behaviours and values’**

#### **CAPACITY 2.1 Engagement between decision-makers and diverse stakeholders and communities, for mutual understanding, appreciation, negotiation and collaboration**

##### Characteristics

- Decision-makers at all levels (community/neighbourhood, city/region, national) engage with diverse actors across all relevant sectors
- Multiple purposes recognised, including mutual understanding and appreciation of lived experience and behaviours, understanding and negotiation of diverse and contested values and visions, and resulting changes in people and/or institutional flexibility and behaviours
- Starts early in the process for issue framing, and is continuing, genuine, inclusive, culturally attuned, long term and relational for trust building, collaboration and co-development approaches

##### FEA process findings

The FEA process confirmed that serious engagement by policy- and decision-makers with urban stakeholders and communities is seen as essential, and at all scales - national, metro/regional, local and citizen/household. Governments should preferably commission such engagement for broader issues, as they should be closest to being ‘owners of the system’ on behalf of the citizens.

Engagement was seen as necessary for understanding of current experience and behaviours, and understanding and negotiation of diverse and contested values and norms; this to inform such activities as collective visioning, framing of significant issues, co-producing knowledge and sharing reflexive learning, co-developing urban directions and solutions with shared commitment, and facilitating inclusive and transparent governance and empowerment.

A number of good practice engagement principles also emerged from the FEA processes based on good and bad experiences at all levels (e.g. starting early and continuing, being genuine, inclusive, culturally attuned, long term and relational for trust building and collaboration). Much current engagement was seen a tokenistic and superficial which is counterproductive.

The engagement challenges for sustainable urban development were seen as being amongst the most complex that exist in participatory research, being spatially and socially diverse, with multiple and contested goals and values.

#### **CAPACITY 2.2 Engagement approaches to be tailored to the context**

##### Characteristics

- Choices to be made along the engagement spectrum (inform, consult, involve, collaborate, empower) according to the issues and purpose
- Choices of engagement tools tailored to issue and participants

##### FEA process findings

Participants considered that the diversity of engagement requirements means that the engagement approach used needs to be contextualized, including positioning along the engagement spectrum (informing-consulting-involving-collaborating-empowering (IAP2 2020)), and using the most appropriate engagement tools and techniques. The FEA process identified many useful tools concluding that for serious engagement more use should be made of collaborative scenario development, deliberative processes, focus groups and urban living labs/local collaborative hubs/visualisation techniques to complement the more common but less personal consultation, submissions and town hall approaches. Participants sought good practice guidance on all the above.

#### **CAPACITY 2.3 Use of boundary spanning intermediaries (e.g. specific issue based, researchers, consultants)**

##### Characteristics

- Includes consultants, researchers, and other actors who emerge around a specific issue
- Seen as independent of decision-makers
- Intermediaries being overt about their values too

##### FEA process findings

Participants recognised the potential for intermediaries (e.g. researchers, consultants, and other actors and networks or bodies emerging on specific issues) to broker boundary-spanning understanding, develop mutual trust, and facilitate collaborative processes. It was also commented that researchers may be more up to date with the latest relevant knowledge, but consultants are often chosen by decision-makers to assist because they more readily identify with the decision-makers context and needs. Whatever the background of intermediaries it was noted that it is important that they also are overt about their own values that they inevitably bring to the process.

### **ENABLER (3) Aligned institutions and governance – ‘The voice of decision-making’**

#### **CAPACITY 3.1 Aligned institutions and coherent policies, plans, resource allocations, finance and decisions across scales, sectors and systems**

##### Characteristics

- Institutions, governance, resource allocations, investment and finance aligned to collectively agreed future directions
- Policy, regulation and planning coherence across sectors, systems, spatial and time scales and jurisdictional/ institutional boundaries
- Institutional innovation so that systemic institutional collaboration opportunities, alignment, and coherence are developed from local to international scales

##### FEA process findings

Participants saw as one of the most critical capacities, the need for greater alignment of institutions with collectively agreed and coherent future directions. The desired collective stewardship included greater coherence of policy, planning, and decision-making across horizontal and vertical organisational and jurisdictional boundaries and across sectors. This implies significant institutional innovation as it requires a clear line-of-sight between federal-state-metro-local governments, whole-of-government approaches across policy areas and sectors, persistence over time based on cross-party political support for sustained urban policies, and deeper understanding of urban systems interdependencies. The potential of the current federal/state/local governments’ coordinated City/Regional Deals approach was noted, but is very much the exception and yet to be proven.

Coherence was also seen as requiring the elimination of perverse funding, performance expectation and incentive practices that encourage current siloed decision-making. Greater coherence would also be evidenced if strategic metropolitan/regional planning and resource allocations clearly reflected collective strategic visions and flexible pathways progressively responding to learning and experimentation (see Capacities 1.1-1.3 above). There was also seen to be a significant imbalance in resource allocations and finance availability as the federal and state governments severely limit and constrain metro and local government funding for local initiatives, even though local government is often best placed to develop local opportunities, engagement and collaborations; and private capital can be reluctant to fund public good benefits from urban innovation and investment.

#### **CAPACITY 3.2 Inclusive, transparent and community-centred formal and informal urban institutions and governance**

##### Characteristics

- Institutional policy and decision-making transparent, accountable and influenced by inclusive and participative governance and engagement processes
- Institutional innovation and structural redesign address formal and informal power imbalances, policies, roles, rules and practices that shape decisions, including giving proper weight to desired common good outcomes
- Institutional redesign reflects both top-down and bottom-up governance roles and initiatives

##### FEA process findings

Participants sought more meaningful inclusion in governance processes, including issue framing, policy and plans development, decision-making, implementation and review. This is partly about the quality of stakeholder and community engagement (see Capacities 2.1-2.3 above), but a more specific ‘governance’ capacity sought, as part of meaningful inclusion, was greater decision-making transparency with evidence that stakeholder, community and expert inputs are seriously taken account of in actual decisions.

Related to this is consideration of *informal* as well as *formal* rules and practices. While there was an emphasis on formal policy, planning and decision-making functions, there was an equal recognition of the need for institutional redesign to address significant informal institutional features (e.g. how individual development decisions stray from formal goals and plans to meet shorter term government, developer and economic interests; conservative cultures and traditional practices and habits/heuristics in urban planning functions; and the importance of building trusted relationships between diverse actors).

There was also recognition that urban transformation needs to reflect both *shared-in-common broader-scale* (top-down) responses and *differentiated local* (bottom-up) governance responses. The FEA process identified many common strategic governance issues that, at least in the Australian context, require new responses at broader scales (e.g. national, state/territory, metro/regional) including

- excessive moves in recent decades towards neoliberal government policies that have promoted private over common good outcomes
- the strategic role of the federal government should be better defined, developed and delivered, including stronger leadership not only in driving urban capacities development, but also working with state governments in multi-level policies, regulations, plans, programs and investments in and across urban related issues such as climate change, energy transitions, water sensitive urban design, transport and housing.
- stronger governance roles at the metro/regional level especially in larger Australian cities, to provide coherence across the multiple local governments
- more actively shaping the critical role of the private sector through a combination of regulation and incentives, and the support of peak industry bodies and larger national developers who often already have quite progressive sustainability views
- more transparent decision-making at all levels, with less political and developer influence, and supported by business cases that reflect wider economic and non-economic values, costs and benefits

At the same time a strong theme was the need for differentiated and better resourced responses at the local level to reflect place-based contexts (e.g. the very different contexts, perspectives and priorities of central city areas, middle suburbs/greyfields, outer city suburbs, peri-urban/rural hinterland areas, and nearby but separate regional centres); as well as local innovative and experimental solutions and governance in conjunction with local communities, citizens, businesses and experts.

#### **CAPACITY 3.3 Critical urban planning capabilities**

##### Characteristics

- Challenge and reinvent strategic urban planning at each level
- Extension of urban planners’ roles and capabilities

##### FEA process findings

Participants saw that the role of urban planners has been degraded vis-a-vis economic agencies and the private sector, and they have been forced increasingly into procedural rather than strategic planning, monitoring and adaptive learning roles. The profession’s peak body (PIA) is fully committed to the need for urban transformational change and was a key participant in the FEA process, nationally and locally. As an example of this there is growing promotion by the PIA and research community of an extension of planners’ capabilities around the potential use of digital transformation to provide broader and more timely perspectives.

From a core position in the multi-form governance they could potentially drive and facilitate many of the necessary changes referred to and also help broker relationships between the multiple stakeholders. This would require reinventing strategic urban planning (e.g. to become scenario based; urban systems, processes and place-based; working across sectors, jurisdictional levels and scales; and linking top-down and bottom-up initiatives). It would also need enhancement of urban planning capabilities including in practising transdisciplinary engagement; using new knowledge, data and technologies; and learning from implementation, research and innovation.

#### **CAPACITY 3.4 Empowered cities, settlements, communities of practice, community groups and individuals**

##### Characteristics

- Empowerment through developing stakeholder and communities’ networks, agency, capabilities and knowledge
- Resources and autonomy for cities, settlements and networked communities to champion change and meet local needs
- A policy environment that supports more sustainable individual citizen and business choices

##### FEA process findings

In the FEA process there was great interest in (and several current examples of) disruptive opportunities, innovative ideas and financing, being combined with local knowledge, networks and capacities to trial, test and govern place-based initiatives meeting local needs (see also Capacities 1.3, 3.5). However centralised funding and power, risk aversion, and reluctance to admit and learn from past mistakes too often work against encouragement of such devolved activities, whether from higher levels of government to city and local governments, or local governments to communities.

Individual businesses and citizens are of course already empowered to make their own personal production, consumption and behavioural decisions and choices which can collectively have a significant impact on sustainability outcomes, and may even drive community networking for certain initiatives (e.g. there was evidence of this in local renewable energy networks). While not yet carried out for a fully representative citizen sample the pilot FEA survey did reveal a strong belief of that particular cohort, that while they did have a personal role in making more sustainable choices, the government has the major levers to make it much easier for individuals to make those choices. So how to influence individual decisions becomes a key governance issue.

#### **CAPACITY 3.5 Institutionally supported innovation and technology facilitation, learning, embedding and acceleration**

##### Characteristics

- Institutional facilitation of distributed urban societal and technological innovation, technology and learning access, experiments and niches
- Institutional facilitation of innovation embedding and acceleration including scaling up and out
- Top down policies, regulation and investment to shape markets and facilitate transformative innovation

##### FEA process findings

The importance of distributed social (including institutional) and technological urban innovation and experimentation for new outcomes and learning was clear (see Capacity 1.3) but it was also clear that in nearly all cases this required institutional support from the public and/or private sectors. Participants also commented on the poor accessibility to information about innovations elsewhere and supportive technologies, and saw the need for institutional capabilities to facilitate leveraging off individual projects for broader impact. Current urban ‘research/knowledge hubs’ or equivalent in Australia provide a very important capability, but are largely focused by sector, issue type, funding source or location, and do not have a mandate or resourcing to take on the broader role.

It was noted that to embed and accelerate successful innovations to transformative change it is important to translate insights and opportunities to higher level urban directions (scaling up) and to broader peer use (scaling out).

Options identified for scaling up from ‘niches’ included top down government resourcing, regulation and market shaping policies typically taking place when the growth of a transformational innovation looks very promising or inevitable. The most frequently raised option was for a network of local urban knowledge hubs to input and share knowledge and innovation insights and opportunities more broadly, as well as to facilitate local knowledge sharing and innovation.

Scaling out can also be aided by inter-city networking and several participants were involved in voluntary local, national and international networks of cities/regions (e.g. C40; Rockefeller 100; ICLEI; GCoM etc).

#### **CAPACITY 3.6 Transformative formal and informal leadership**

##### Characteristics

- Leadership can come from all sectors - governments, private sector, professions and communities
- Formal leadership can mainstream comprehensive sustainable urban development into institutional roles
- Formal and informal leadership can articulate new narratives, bridge barriers and differences, and motivate engagement and collaboration

##### FEA process findings

The FEA process identified stronger and more consistent federal and state government leadership as crucial, but also the need for local champions and leaders who may more often have informal roles. Formal leadership roles identified included policy and practice leadership and mainstreaming urban sustainable development capacities into institutional roles, reform and redesign. Formal and informal leadership included articulating shared visions, motivating engagement, and shaping collaborative and relational processes. Examples of strong private sector, professions and NGO leadership were also noted, often from larger and better resourced corporations and peak bodies.

### **ENABLER (4) Knowledge co-production, usage and learning – ‘The voice of expertise’**

#### **CAPACITY 4.1 Co-produced, shared and used knowledge**

##### Characteristics

- Co-developed mission-oriented research agendas shape priorities and foster research and user collaboration
- Transdisciplinary co-production of knowledge enhances relevance, credibility and uptake
- Shared knowledge and data platforms and brokers facilitate integrated, accessible, translated and used knowledge

##### FEA process findings

In the FEA process a broad view was taken of knowledge to include experience, information, data, analysis, modelling, evidence (translated knowledge), and innovations/ technology/ solutions (applied knowledge). There was a strong belief that good *existing* knowledge and credible expertise is often available to support evidence-based decision-making, but not known about or overridden by political and private sector interests. Increasing co-production of new knowledge (starting with co-design and issue framing) and better facilities for knowledge sharing, were supported to improve uptake of both new and existing knowledge that is relevant, credible, integrated, accessible and translated. Again, local and networked knowledge hubs were seen as a potential solution.

Excellent research activities and centres of urban expertise in Australia were identified, many already using exemplary co-production approaches. Individually they tend to be sector or issue based, but collectively they cover a broad range of urban-relevant disciplines. However, many have limited life funding and none currently has the charter, program or funding to develop a more integrated systems view of urban development or to provide an ongoing synthesis of and access to the broader range of urban knowledge. Consequently, research initiation and knowledge access are highly fragmented with little collaboration on broader national, state or city research agendas, unconnected knowledge holdings and platforms, and a funding environment that mostly encourages competition rather than collaboration.

In respect of urban data AURIN provides a national coordination capability, but advised that it’s role could be significantly enhanced with more open access to data sources, the rapidly increasing new streams of ‘smart city’ data and technologies (e.g. metadata, APIs, AI/ ML/visualisation tools, spatio-statistical tools, and spatio-temporal predictive models), and links to other sectoral data platforms. Apart from AURIN in respect of data, there is no national approach to urban knowledge agenda setting or access, even though the most recent government supported Australian National Outlook identified shift in urban development as one of five major national shifts required for the country to shift to a more desirable and sustainable trajectory (the other four being land use, energy, industrial composition, and culture).

The FEA process provided some insight to the range of relevant research and knowledge areas that might underpin an emerging ‘urban science’ or ‘science of cities’ and research agendas (see Fig. 3 in main article) and several international examples were identified. This included the JPI Urban Europe SRIA 2.0 urban research agenda for the EU. This was of particular interest as it moves towards an integrated systems’ view by being ‘mission-focused’ as also suggested by the FEA process (see Capacity 4.3 below).

#### **CAPACITY 4.2 Diverse knowledge sources and disciplines**

##### Characteristics

- Diverse sources based on all stakeholders, communities and experts being both providers and users of knowledge
- Multiple disciplines and methods employed in making sense of the knowledge

##### FEA process findings

The FEA process included the widest possible range of participants as it started from the premise that all stakeholders and communities should be seen as both knowledge providers and knowledge users, and that urban knowledge systems extend well beyond researchers. The participants clearly supported the transdisciplinary and collaborative approaches. This extended to the range of expert input required with recognition that multiple disciplines and methods are also needed to provide insights on complex urban systems and actionable knowledge.

#### **CAPACITY 4.3 Urban systems awareness, knowledge and cumulative understanding**

##### Characteristics

- Capabilities to analyse, understand and model complex urban systems, subsystems and cross-sector nexus
- Cumulative systems and nexus insights increasingly inform urban visioning, engagement and governance processes
- Whole-of-system ‘urban science’ shapes framing of urban challenges, missions and associated research/knowledge agendas, co-production and platforms

##### FEA process findings

The FEA process, being cross-sector and cross-disciplinary, evidenced the need for supporting knowledge to be systems-based (across urban processes, sectors, disciplines and scales - space and time). This was seen as necessary to help understand and support broader issues framing and analysis, to help break down traditional institutional and disciplinary siloes, and thus also help the development of shared visioning, research agendas, knowledge platforms, and trade-offs and synergies for specific urban decisions. However, in the context of a specific urban context or issue, a systems’ approach also requires greater understanding of the relevant path dependencies, interdependencies, systems dynamics and emergence, underlying patterns, and systems leverage points; and doing this across spatial and time scales.

Understanding and accumulating knowledge on the whole urban system were seen as very challenging, but useful steps can be taken through nexus thinking where highly interconnected subsystems are considered jointly (e.g. land use and transport, food-energy-water connections and joint climate change mitigation and adaptation examples were identified). Insights into urban systems can also be supported by modelling approaches that capture major systems interdependencies and feedbacks.

#### **CAPACITY 4.4 Policy-practice-research capabilities and collaborations**

##### Characteristics

- Policy-practice-research collaboration, interchange and mutual skills development
- Improved issue framing; knowledge co-production and uptake; and collective reflexivity and learning
- Communities of practice and knowledge brokering develop at multiple levels

##### FEA process findings

Participants recognised a need to continually develop the capabilities of policy-makers and practitioners in research/knowledge commissioning, access and usage, and the capabilities of researchers to better understand and engage with policy and decision-making environments. This included capabilities in collaborative engagement and issue framing; knowledge co-production, interpretation, dissemination and translation; and collective self-assessment, reflexivity and social learning; all supported by improving the range of policy-practice-research interfaces and activities. While there are examples of excellent collaboration within and outside Australia, there is little institutional support for developing the sorts of capability-building required by the other proposed capacity building initiatives. Several potential initiatives were identified for the National Strategy.

#### **CAPACITY 4.5 Reflexivity and learning**

##### Characteristics

- Collective transdisciplinary self-assessment for social learning and feedback into goals, policy, strategy, plans, processes, research and capacities development at multiple levels

##### FEA process findings

The diversity of participation especially in the workshops evidenced the necessity and feasibility of listening carefully to, and being prepared to learn from, others. The co-design framework which informed the design of the FEA process (Fig. 1 of the main article), explicitly built in reflexivity and learning into both research and practice, and the FEA strategy co-development process was itself an example of iterative and reflexive learning. More broadly it was recognised that such a capacity should draw on formal monitoring of urban development performance/outcomes, but also reflection on deeper implications that challenge current research and practice paradigms in use.
