# Supplementary material for: Enabling urban systems transformations: co-developing national and local strategies
Source: Urban Transform. 2023 Feb 20;5(1):5. doi: 10.1186/s42854-023-00049-9 (PMC9939254; doi:10.1186/s42854-023-00049-9)
Supplement: Supplementary file 3 — Additional file 3. Mapping of other published ‘sustainability’ and ‘urban’ systems transformation capacity frameworks to the EUST framework enablers and capacities (EUST framework at Fig. 2 and Table 2 in main article text). [file 42854_2023_49_MOESM3_ESM.docx]

## Additional file 3 Mapping of other published ‘sustainability’ and ‘urban’ systems transformation capacity frameworks to the EUST framework enablers and capacities (EUST framework at Fig. 2 and Table 2 in main article text)

Thirteen articles are included in the tabular comparison below with the EUST framework (Fig. 2 and Table 2 in the main article). All frameworks are about intentional and transformational change for sustainability, and guidance as to capabilities necessary to pursue this. Six are specifically in an urban context and the others more generally relate to transformation of social-ecological systems (SES) or equivalent. This distinction does not seem to be material as urban settlements are amongst the most complex of SES. The descriptions in each enabler’s column are from the related publication.

Comparison shows that, although they each come from somewhat different perspectives, all the frameworks can be mapped quite well to the EUST ‘4 enablers’ framework (Fig. 2 in the main article). The mapping is never perfect because each perspective has its own emphasis, uses somewhat different terminology and sometimes slightly different grouping of underpinning concepts. However, it is clear that they are all describing essentially the same ‘transformation enabling’ paradigm. Where there is more detail of the underpinning ‘capacities’ (or equivalent), they also can be matched to one or more of the underpinning capacities identified in the EUST framework (Table 2 in the main article).

The intent is not to take away from the independent value of each published article – there is clear value in the different perspectives. However, at the same time, in order to make the research more convergent, cumulative and useful to policy-makers and practitioners, there is also value in showing how they can be related to an overarching transformation enabling paradigm. They should be seen as complementary and potentially cumulative rather than competing views.

### Table Af3 Mapping between ‘EUST’ framework’s four transformation enablers, and enablers (or equivalent) in other studies

| **Mapping between the ‘EUST’ framework’s four transformation enablers, and enablers (or equivalent) from other studies** | | | |
| --- | --- | --- | --- |
| **ENABLER 1.**  **Co-evolutionary design and navigation**  ***‘The voice of intent’*** | **ENABLER 2.**  **Engagement between decision-makers, stakeholders and communities**  ***‘The voice of experience, behaviours and values’*** | **ENABLER 3.**  **Aligned institutions and governance**  ***‘The voice of decision-making’*** | **ENABLER 4. Knowledge co-production, usage and learning**  ***‘The voice of expertise’*** |
| **(1) Wolfram (2016) on ‘Conceptualising urban transformative capacity: A framework for research and policy’**  Identifies 10 capacities (C1 – C10; some with sub-capacities) in a conceptual framework for developing ‘urban transformative capacity’ in pursuit of urban sustainability, drawing on contributions from a range of publications on societal systems or ‘subject areas’. Wolfram et al. (2019) summarise application of the framework to a number of international case studies in the Global North and South, which identifies some recurring emphases on certain capacities (e.g. inclusion/empowerment, roles of intermediaries, strategic urban planning, leadership, reflexive social learning) | | | |
| **C5 Sustainability**  **Foresight**  (normative  participatory visioning and alternative scenario designs)  **C5.2 Collective vision for radical sustainability changes**  **C5.3 Alternative scenarios and future pathways**  **C6 Experiments C6** (practical experimentation of communities with novel  solutions) plus:  **Innovation (**Create, nurture and anchor novelties)  **Exnovation (**Expose and dismantle  path-dependencies) | **C9 Agency levels C9 (**individuals, households, social groups, organizations, networks, society, institutions roles in diverse relational engagement and contestation, working across multiple human agency levels for social justice)  **C1.3 Sustained intermediaries and hybridization** | **C1 Inclusive & Multiform Urban Governance (**broad stakeholder involvement, diversity of formal and informal interactions, as well as effective intermediation)  **C1.1 Participation and inclusiveness**  **C1.2 Diverse governance modes and network forms**  **C2 Transformative Leadership C2 (**lead through articulating shared visions, motivating engagement and shaping collaborative processes)  **C3 Empowered Communities of Practice (CoP) (**how far resources are accessible and conditions of autonomy provided for such communities to meet social needs - place based and/or issue driven)  **C3.1 Addressing social needs and motives**  **C3.2 Community empowerment and autonomy**  **C7 Innovation Embedding C7 (**e.g., through resource provision or regulations  **C7.1 Access to resources for transformative capacity building**  **C7.2 Planning and mainstreaming transformative action**  **C7.3 Reflexive and supportive regulatory frameworks**  **C10 Scale levels** (collective stewardship to enable and align diverse actions across political-administrative levels and geographical scales - site, neighbourhood, district, city, region, nation, inter- and transnational) | **C5.1 Diversity and transdisciplinary co-production of knowledge**  **C4 System(s) Awareness (**systems analysis to understand change dynamics and path dependencies)  **C4.1 Baseline analysis and system(s) awareness**  **C4.2 Recognition of path dependencies**  **C8 Reflexivity and Learning** (Social learning feeds outcomes of all four processes (C4-7) back into the articulation of agency i.e. governance,  leadership, and community empowerment (C1–3)) |
| **(2) Waddell et al. (2016) on ‘Societal Change Systems: A Framework to Address Wicked Problems’**  Identifies 7 Societal Change Functions (SCFs) to address ‘the how’ question in tackling wicked problem/complex adaptive systems issues; developed with UN–supported SE4All initiative (or SEFA), in context of delivering on SDG 7 energy, but SCFs considered generic to wicked problems; draws on resilience, SES and socio-technical transitions (STT) thinking. | | | |
| **Visioning** (generate shared understanding and vision)  **Prototyping (**developing examples of the future) | **Organising** (bringing together diverse stakeholders to generate coherence in strategies and creation of legitimacy)  **Advocating** (Mobilizing voice and increasing pressure upon specific stakeholders who are  blocking (actively or inactively) change) | **Resourcing** (providing financial/people resources)  **Assessing** (developing indices, assessments, and/or certification processes) | **Learning, research, capacity development**  (developing and disseminating new  knowledge and tools with research, piloting  new approaches, and training) |
| **(3) Scoones et al. (2019) on ‘Transformations to sustainability: combining structural, systemic and enabling approaches’** Identifies 3 cross cutting ‘enabling’ principles to progress complementary structural, systems (SES/STT) and process enabling approaches to address societal transformations to sustainability and the SDGs transformation. Argues that structural and systemic approaches need to be ultimately underpinned by enabling approaches, which emphasises processes and capacities rather than just outcomes. | | | |
| **Selecting between plural pathways** (plurality of ways to get towards multiple — very material — institutional and infrastructural transformations, reflecting different ideas and values of sustainability) | **Taking plural pathways seriously** (new forms of deliberation/engagement amongst contending actors, not just with diverse ideas, but also with the contrasting norms, interests and practices.) | **Taking politics seriously** (political nature of transformation - however well assisted by technical expertise — engaging with a diversity of contexts or a plurality of perspectives is always deeply political; and wider political institutions, economic systems and technical infrastructures shape outcomes and possibilities; so will need to confront disparate views, interests and forms of incumbent power). | **Taking diverse knowledges seriously** (requires transdisciplinarity, where multiple forms of expertise co-construct new knowledges that are both broader in what they consider and more open in their implications for change; in turn requires more equal processes of collaboration and exchange, exploring diverse visions from different standpoints; ‘transformation labs’ have been used in a number of recent initiatives as spaces for dialogue around transformation) |
| **(4) Abson et al. (2017) on ‘Leverage points for sustainability transformation’**  Identifies three high leverage transformational ‘sustainability interventions’ in social-ecological systems of interest (i.e. reconnecting people to nature, restructuring institutions, and rethinking how knowledge is created and used). Draws on Meadows (1999) hierarchy of systems leverage points, and groups the highest levels as representing systems ‘intent’ and systems ‘design’ – the three high leverage interventions summarised above contribute to each of these. | | | |
| **Intent and design: the emergent direction to which a system of interest is oriented** (arising from the multiple, potentially conflicting, sets of world views, goals and purposive behaviours within a given system of interest). | **Intent contribution:** the underpinning values, worldviews of actors  **Design contribution:** social structures.  **Leverage intervention 1: Reconnect** -people’s connections  to nature and their influences on sustainability outcomes | **Intent contribution**: the underpinning values, worldviews of actors  **Design contribution**: institutional structures of rules, power and self-organisation.  **Leverage intervention 2: Restructure** - the role of institutions and institutional decline and failure in systemic change | **Intent contribution**: the underpinning values, worldviews of actors  **Design contribution**: Structure of information flows  **Leverage intervention 3: Rethink** - knowledge production and use in  transformational processes |
| **(5)** **Moser et al. (2019) on ‘Transformations’**  Identifies 6 conditions of systems change reflecting various levels of transformational leverage and change (linked to Meadows (1999) 12 leverage levels). Transformative actions can be taken at all levels and in ways that address the root causes of complex systems issues like climate change, social vulnerability and societal challenges, helping to achieve mitigation and adaptation goals as well as the SDGs. Power dynamics is a critical issue, requiring (e.g.) courageous leadership, bottom up as well as top down action, national planning frameworks. | | | |
| **Intent (Levers 1-3)**  (e.g. Redefining national self-interest) | **Mindsets (Levers 1-3**) (e.g. values and worldviews)  **Relationships, connections (Levers 7-9) (**e.g. Dialogic spaces for shared values, including across social divides)  **Procedural (Levers 10-12)**  **Practices** (e.g. Transformation Labs (T-Labs) where stakeholders listen, learn, vision, identify shared values, and explore transformative pathways) | **Power dynamics (Levers 4-6)**  (e.g. Courageous leadership; Top down (goal setting, enabling) and bottom up; National planning frameworks)  **Procedural (Levers 10-12)**  **Policies**  (e.g. Economic policies, sector strategies; Acceleration of technology change; Strategic, context-sensitive territorial planning at subnational levels; Diversification of livelihoods)  **Practices** (e.g. Giving self-organized associations at the local level, e.g., agricultural or fishermen’s organizations, decision-making authority to design transformative pathways)  **Resource flows** |  |
| **(6) Gorddard et al. (2016) on ‘Values, rules and knowledge: Adaptation as change in the decision context’**  Identifies values, rules and knowledge (vrk) framework as co-evolving drivers of ‘decision-context’ for more transformational adaptation of complex societal and ecological systems responding to impacts of global change (e.g. climate adaptation). The vrk approach informs and shapes the agency-structure nexus – vrk changes the structure (institutional units) which then conditions agency and decision-making | | | |
| **Co-evolutionary adaptation and change between societies and nature** (including co-evolution in the intertwined Values, Rules and Knowledge (vrk)) | **Values** (ethical precepts/moral principles/preferences/desirable goals that determine the way people select actions and evaluate events, so can be used to assess decision options) | **Rules** (provide agreed guidance on how to behave and enable implementation. Includes rules-in-use (norms, practices, habits, heuristics) and rules-in-form (regulations, laws, directives) The decision-making (and agency) perspective, which is enabled or legitimised by current vrk) | **Knowledge** (of options and their implications. Includes evidence-based (scientific and technical) knowledge and experiential knowledge.) |
| **(7) Beddoe et al. (2009) on ‘Overcoming systemic roadblocks to sustainability’**  Identifies worldviews, institutions, and technologies (WIT) framework for evolutionary redesign of social-ecological systems regime and/or culture to achieve sustainability. Aim is co-evolution towards intent as smoothly as possible**.** | | | |
| **Evolutionary redesign and selection** of cultural variants through learning, as cultural change and evolution act on and respond to the Worldviews, Institutions and Technologies | **Worldviews as** unconscious assumptions and perceptions of how the world works. These unconscious assumptions about how the world works provide the boundary conditions within which institutions and technologies are designed to function. | **Institutions as** cultures’ rules and norms. Key structures that are universal among all cultures: kinship, economy, religion, polity, governance, and education (21). These structures constrain individuals’ behaviour, define a recognizable culture (18), and serve as problem-solving entities that allow societies to adapt to their environments (21–23). | **Technologies** as applied information/ knowledge. Technologies are broadly defined as the applied information that we use. |
| **(8) Grimm et al. (2000) ‘Integrated approaches to long-term studies of urban ecological systems’**  Identifies three fundamental drivers of the human elements of urban and peri-urban ecosystems (culturally based values and perceptions; institutions and organisations; flow of information and knowledge). The article is primarily about understanding the complex urban ecological systems rather than how to identify shared intent. | | | |
|  | **Culturally based values and perceptions** | **Institutions and organisations** | **Flow of information and knowledge** |
| **(9) Kangas et al. (2019) on ‘Leading Change in a Complex World: Transdisciplinary Perspectives’**  Identifies three ‘ideas’ (dealing with complexity, relationality, and dynamic change) as necessary capabilities in a ‘leadership for change’ framework i.e. relational leadership and emergent change needed for complex systems. Leadership needs to understand full range of underpinning capacities needed, including dispersed power, bottom up as well as top down action, ‘chiasmatic’ (intersecting) relational and entity-based leadership dynamics. The focus is on the characteristics of transformational change that leadership needs to handle rather than all the capacities needed for change but still provides an interesting perspective to map into the framework. | | | |
| **Idea 1**: Complex - Many contemporary change processes are complex and systemic so emergent rather than planned change | **Idea 2**. Relational - Issues and problems crossing traditional institutional boundaries cannot be resolved by a single actor or sector so governments, businesses, civil society actors, citizens and consumers must work together, negotiate, and agree on novel ways of doing things; need to understand how different societal sectors and actors function and make sense of the world, and how they can work together. | **Idea 1**: Complex - Many contemporary change processes are complex and systemic so need capacity to think across traditional institutional boundaries. Requires relational not hierarchical/entity leadership. | **Idea 1**: Complex - Many contemporary change processes are complex and systemic so need the ability to see patterns and structures beyond specific elements and events.  **Idea 3**: Dynamic change - Complex environments are marked by ambiguity and uncertainty so need the capacity to embrace uncertainty and use various sources of information and knowledge to create a shared understanding of the situation at hand. |
| **(10) Iwaniec et al. (2019) on ‘The Framing of Urban Sustainability Transformations’**  Identifies seven criteria for framing of transdisciplinary research for intentional urban sustainability transformation. Transdisciplinary/co-produced research and action that addresses all the capacities, is a necessary but not sufficient condition for transformation. Notes that need to consider other (non-research) factors e.g. local governance, available resources, researcher-practitioner networks to action towards transformation. This article, while still relevant to the comparison has a focus on what constitutes urban transformation rather than the capacities needed to deliver that transformation. It therefore informs rather than defines the capacities. | | | |
| **Fundamental** (radical and desirable state or regime change including in social/behavioural and governance systems and path dependency)  **Persistent** (long term pursuit of future trajectories/ outcomes; including building ongoing capacities)  **Open ended** (multiple, branched, emergent and adaptive pathways | **Normative** (incorporate just and desirable values) |  | **Co-produced** (collaborative inclusive endeavour)  **Evidence based** (critical and pluralistic diverse forms and sources of knowledge)  **System based** (holistic representation of the  interdependent components across sectors, space and time) |
| **(11) H**ö**lscher et al. (2019) on ‘Tales of transforming cities: Transformative climate governance capacities’**  Identifies four governance capacities for transformative climate governance – stewarding, unlocking, transformative and orchestrating capacities. Although for climate governance, these could readily be extended to more general urban sustainable development. The framework comes primarily from the perspective of capacities required of the institutions with the most substantial urban decision-making power and is therefore useful in the detail it provides in that context. It could therefore in principle all be mapped into Column 3 (i.e. Enabler 3 the ‘Voice of decision-making’). However, because it is concerned with the institutional governance capacities to enable transformation, it also usefully relates to capacities required for the other three EUST framework transformation ‘enablers (or voices)’ where it primarily reflects the required roles of decision makers in those contexts. To some extent the social/network, institutional and knowledge/network ‘conditions’ in the framework reflect Enablers 2,3,4 respectively, and many of the components reflect capacities needed to develop Enabler 1, the Voice of Intent, though the match is not perfect. The mapping below draws on the detail in the Hölscher et al. (2019) framework, and shows that some components relate to more than one column. | | | |
| **->1.2 Strengthening self-organisation for stewarding** (***Network condition: Multi-scale and cross-sectoral***  ***networks and partnerships for risk planning and***  ***management***: Establishing issue-specific, multi-level and cross-sectoral collaborations to develop and implement projects in line with context needs; Involving communities in joint and context-specific visioning, planning and implementation processes)  **->2.1 Strategic alignment**  (***Institutional condition: Long-term and integrated goals***: Developing long-term climate mitigation and adaptation, sustainability and resilience goals)  **3. Transformative capacity**  **3.1 Enabling novelty creation (also->Col 2 and Col 3)**  ***(Social condition: Leadership for creating and using opportunities for change:*** Mobilising political leadership to put new and ambitious goals on the agenda; Making use of momentum and opportunities for change; Piggy-backing and quickly expressing potential of a new solution)->Col3  (***Network condition: Multi-actor innovation networks:*** Forming informal ‘coalitions of the willing’ for strategic and operational innovation; Involving communities in design and implementation of experiments)->Col2  (***Institutional condition: Regulatory, financial space for innovation***: Temporary lifting or avoiding existing regulations)->Col3  **3.2 Increasing visibility of novelty (also-> Col2 and Col4)**  **(*Social condition: (Trans)- local support for the innovation story:*** Creating and advocating an inspiring innovation story; Showcasing innovations as market potential for the city)->Col2  **(*Network condition: Advocacy*** c***oalition***s: Creating advocacy coalitions to carry the innovation story: Participating in and hosting local, regional, national and international  networking, best practice and knowledge exchange events for visibility)->Col2 and Col4  **3.3 Anchoring novelty in context (also->Col 3 and Col 4)**  (***Knowledge condition: Learning for replication and***  ***upscaling:*** Identifying proof-of-concept lessons from innovations to facilitate  replicating and embedding;  Identifying opportunities from innovation for upscaling;  Identifying bricolage of solution elements to mainstream innovations into  urban planning processes and decisions)->Col3 and Col4  ***(Network condition: Self-sustaining innovation networks***: Formalising operational public-private partnerships for continuous  innovation; Setting up cross-sectoral networks and partnerships tasked with  (embedding of) innovation in institutional structure)->Col3  (***Institutional condition: Institutional space for embedding strategic and operational innovations in mainstream practice:*** Creating open mind-set for taking up innovations in tactical agendas and daily practices; Allocating budget to developing and maintaining innovation, upscaling and replicating)->Col3  **4. Unlocking capacity**  **4.1 Revealing unsustainable path-dependency and mal-adaptation (also->Col4)**  (***Knowledge condition: Identifying and exploring systemic drivers***: Identifying systemic social and economic drivers of unsustainability and  path-dependency; Road mapping and scenario analyses to explore phase-out options; Conducting regular emissions inventories)->Col4  (***Network condition: Knowledge partnerships*:** Establishing public-private knowledge partnerships to identify drivers and  explore phase-out options)->Col4  (***Institutional condition: Knowledge mandates:*** Mandating knowledge generation to ensure access to data)->Col4  **4.2 Undermining vested interests and incentive structures (also->Col3)**  ***(Institutional condition: Support for sustainable business cases and investments:*** Setting standards for sustainable investments;  Providing incentives for sustainable investments;  Integrating sustainability into public tendering)->Col3  (***Institutional condition: Control of unsustainable practices***: Implementing regulation to control unsustainable practices)->Col3  **4.3 Breaking open resistance to change (also->Col2 and Col3)**  **(*Social condition: Societal and political awareness and***  ***support:*** Raising awareness and providing assistance for sustainable investments  and behaviour change; Lobbying for political support)->Col2 and Col 3  ***(Network condition: Key support networks and***  ***Partnerships:*** Setting up public-private partnerships for issue-specific action; Setting up support networks with key stakeholders’ groups)->Col3 | **->1.2 Strengthening self-organisation for stewarding**  (***Network condition: Multi-scale and cross-sectoral networks and partnerships for risk planning and management***: Establishing issue-specific, multi-level and cross-sectoral collaborations to develop and implement projects in line with context needs; Involving communities in joint and context-specific visioning, planning and implementation processes)  (***Social condition: Social capital and actor empowerment:*** Raising awareness about risks and response options; Strengthening social networks to enable self-organised response and social resilience)  **->2.1 Strategic alignment**  (***Social condition: Involvement of multiple actors in shared strategy formulation and visioning***: Involving multiple actors from different city departments and private organisations in strategy formulation; Public outreaching and participation)  **->3.1 Enabling novelty creation**  ***(Network condition: Multi-actor innovation networks:*** Forming informal ‘coalitions of the willing’ for strategic and operational innovation; Involving communities in design and implementation of experiments)  **->3.2 Increasing visibility of novelty**  **(*Social condition: (Trans)- local support for the innovation story:*** Creating and advocating an inspiring innovation story; Showcasing innovations as market potential for the city)  **(*Network condition: Advocacy*** c***oalition***s: Creating advocacy coalitions to carry the innovation story: Participating in and hosting local, regional, national and international  networking, best practice and knowledge exchange events for visibility)  **->4.3 Breaking open resistance to change**  **(*Social condition: Societal and political awareness and support:*** Raising awareness and providing assistance for sustainable investments  and behaviour change; Lobbying for political support) | **1. Stewarding capacity**    **1.2 Strengthening self-organisation for stewarding (also**  **->Col1 and Col2)**  ***(Institutional condition: Flexible, problem-based and fit-to context planning and management approaches***: Integrating long-term, systemic risks and uncertainties into planning and management approaches; Adopting problem-based, fit-to-context and no-regret planning and management approaches; Providing flexible regulation and incentives to facilitate fit-to-context risk protection; Clearly assigning and communicating responsibilities of actors)  (***Network condition: Multi-scale and cross-sectoral***  ***networks and partnerships for risk planning and***  ***management***: Establishing issue-specific, multi-level and cross-sectoral collaborations to develop and implement projects in line with context needs; Involving communities in joint and context-specific visioning, planning and implementation processes) ->Col1 and Col2  (***Social condition: Social capital and actor empowerment:*** Raising awareness about risks and response options; Strengthening social networks to enable self-organised response and social resilience) ->Col2  **1.3 Monitoring and continuous learning (also ->Col4)**  (***Knowledge condition: Institutional and social memory:*** Drawing on past experience and learning about new solutions; Continuously updating plans and resilience and sustainability indicators) ->Col 4  **2. Orchestrating capacity**  **2.1 Strategic alignment (also -> Col 1and Col 2)**  (***Institutional condition: Long-term and integrated goals***: Developing long-term climate mitigation and adaptation, sustainability and resilience goals)->Col1  (***Social condition: Involvement of multiple actors in shared***  ***strategy formulation and visioning***: Involving multiple actors from different city departments and private organisations in strategy formulation; Public outreaching and participation)->Col2  **2.2 Mediation across scales and sectors-(also->Col4)**  ***(Network condition: Connection nodes for pooling climate***  ***action:*** Establishing central connection nodes for pooling climate efforts at multiple levels; Establishing cross-departmental city offices for coordinating and  knowledge brokering at multiple levels; Designating theme-leads and contact persons within individual departments)  (***Network condition: Intermediary spaces for knowledge***  ***sharing and trust building:*** Creating neutral co-creation spaces and knowledge partnerships to build trust for knowledge sharing and resource synergies across scales and  sectors; Participating in international city networks; Establishing cross-departmental co-creation spaces for knowledge exchange, priority alignment and trust building)->Col4  (***Knowledge condition: Pooling and integrating knowledge***  ***and resources across scales and sectors:*** Identifying opportunities, synergies and trade-offs between different goals)->Col4  **2.3 Creating opportunity contexts**  (***Institutional condition: Framework conditions and***  ***financing mechanisms for long-term co-benefits***: Redefining responsibilities for carrying costs; Creating competitions to leverage innovative, long-term and co-beneficial solutions)  **->3.1 Enabling novelty creation**  ***(Social condition: Leadership for creating and using opportunities for change:*** Mobilising political leadership to put new and ambitious goals on the agenda; Making use of momentum and opportunities for change; Piggy-backing and quickly expressing potential of a new solution)  (***Institutional condition: Regulatory, financial space for innovation***: Temporary lifting or avoiding existing regulations)  **->3.3 Anchoring novelty in context**  (***Knowledge condition: Learning for replication and***  ***upscaling:*** Identifying proof-of-concept lessons from innovations to facilitate  replicating and embedding;  Identifying opportunities from innovation for upscaling;  Identifying bricolage of solution elements to mainstream innovations into  urban planning processes and decisions)  ***(Network condition: Self-sustaining innovation networks***: Formalising operational public-private partnerships for continuous  innovation; Setting up cross-sectoral networks and partnerships tasked with  (embedding of) innovation in institutional structure)  (***Institutional condition: Institutional space for embedding strategic and operational innovations in mainstream practice:*** Creating open mind-set for taking up innovations in tactical agendas and daily practices; Allocating budget to developing and maintaining innovation, upscaling and replicating)  **->4.2 Undermining vested interests and incentive structures**  ***(Institutional condition: Support for sustainable business cases and investments:*** Setting standards for sustainable investments;  Providing incentives for sustainable investments;  Integrating sustainability into public tendering)  (***Institutional condition: Control of unsustainable practices***: Implementing regulation to control unsustainable practices)  **->4.3 Breaking open resistance to change**  **(*Social condition: Societal and political awareness and***  ***support:*** Raising awareness and providing assistance for sustainable investments  and behaviour change; Lobbying for political support)  ***(Network condition: Key support networks and***  ***Partnerships:*** Setting up public-private partnerships for issue-specific action; Setting up support networks with key stakeholders’ groups) | **1.1 Generating knowledge about system**  **dynamics**  (***Knowledge condition: Long-term, systemic and context specific knowledge about risks and uncertainties***: Long-term forecasting of systemic risks and uncertainties across scales; Generating problem-based and context-specific knowledge in vulnerability hot spots; Identifying and prioritising high-risk areas for directing investments)  ***(Network condition: Knowledge partnerships***: Creating issue-specific and multi-stakeholder research programmes and partnerships for knowledge generation across scales and sectors; Formalising research partnerships and networks)  ***(Institutional condition: Knowledge mandates:*** Mandating knowledge generation to ensure access to data)  **->1.3 Monitoring and continuous learning**  (***Knowledge condition: Institutional and social memory:*** Drawing on past experience and learning about new solutions; Continuously updating plans and resilience and sustainability indicators)  **->2.2 Mediation across scales and sectors**  (***Network condition: Intermediary spaces for knowledge***  ***sharing and trust building:*** Creating neutral co-creation spaces and knowledge partnerships to build trust for knowledge sharing and resource synergies across scales and  sectors; Participating in international city networks; Establishing cross-departmental co-creation spaces for knowledge exchange, priority alignment and trust building)  (***Knowledge condition: Pooling and integrating knowledge***  ***and resources across scales and sectors:*** Identifying opportunities, synergies and trade-offs between different goals)  **->3.2 Increasing visibility of novelty**  **(*Network condition: Advocacy*** c***oalition***s: Creating advocacy coalitions to carry the innovation story: Participating in and hosting local, regional, national and international  networking, best practice and knowledge exchange events for visibility)  **->3.3 Anchoring novelty in context**  (***Knowledge condition: Learning for replication and***  ***upscaling:*** Identifying proof-of-concept lessons from innovations to facilitate  replicating and embedding;  Identifying opportunities from innovation for upscaling;  Identifying bricolage of solution elements to mainstream innovations into  urban planning processes and decisions)  **->4.1 Revealing unsustainable path-dependency and mal-adaptation**  (***Knowledge condition: Identifying and exploring systemic drivers***: Identifying systemic social and economic drivers of unsustainability and  path-dependency; Road mapping and scenario analyses to explore phase-out options; Conducting regular emissions inventories)  (***Network condition: Knowledge partnerships*:** Establishing public-private knowledge partnerships to identify drivers and  explore phase-out options)  (***Institutional condition: Knowledge mandates:*** Mandating knowledge generation to ensure access to data) |
| **(12) Shahani et al. (2021) on ‘Transformative low-carbon urban innovations: Operationalizing transformative capacity for urban planning’.**  Uses Wolfram (2016) urban transformation capacity framework (C1 – C10), which therefore maps to enablers the same way as does table entry (1) above for that study. However, it extends the detail within this to ‘dimensions’ and related urban planning capacities needed in the context of people/social- and nature/ecological-based low-carbon urban solutions. The ‘dimensions’ are identified from a literature review and are shown in italics in square brackets below, mapped to the Wolfram (2016) framework. | | | |
| **C5 Sustainability**  **Foresight**  (normative  participatory visioning and alternative scenario designs) *[Knowledge co-production is*  *organized with expertise and with the expectation or prospect of scale out participation over time; Transdisciplinary knowledge to map out forecasted benefits across sectors; Alternative pathways include a mix of disruptive and*  *conforming/incremental low-carbon innovations that co-shape the route to*  *radically alternative futures]*  **C5.2 Collective vision for radical sustainability changes**  **C5.3 Alternative scenarios and future pathways**  **C6 Experiments C6** (practical experimentation of communities with novel  solutions) plus:  **Innovation (**Create, nurture and anchor novelties)  **Exnovation (**Expose and dismantle  path-dependencies)  [*Experiments need to be developed through a coproduction/ co-design process centring on social learning and coordination of activities across different actors and scales]* | **C9 Agency levels C9 (**individuals, households, social groups, organizations, networks, society, institutions roles in diverse relational engagement and contestation, working across multiple human agency levels for social justice) *[ Engaging citizens,*  *communities, marginalized groups, and NGOs; Collaborating between governments and civic society; Involving policymakers, planners, and politicians]*  **C1.3 Sustained intermediaries and hybridization** | **C1 Inclusive & Multiform Urban Governance (**broad stakeholder involvement, diversity of formal and informal interactions, as well as effective intermediation) *[Diversity, inclusivity, and activation of urban actors; Self-governance arrangements of urban projects; Urban experiments are settings of inclusive governance]*  **C1.1 Participation and inclusiveness**  **C1.2 Diverse governance modes and network forms**  **C2 Transformative Leadership C2 (**lead through articulating shared visions, motivating engagement and shaping collaborative processes) [*Shift to bottom-up or local leadership in uprooting new ideas, technologies, and approaches; Thematically focussed initiatives; Connects initiatives and opportunities across* *(multi-level) governance scales*]  **C3 Empowered Communities of Practice (CoP) (**how far resources are accessible and conditions of autonomy provided for such communities to meet social needs - place based and/or issue driven) *[Social needs and motives need to be addressed through*  *inclusive urban design; Recognize equity as a value and a guiding principle; Social learning is a vehicle for empowering communities; Equip communities so as to sustain empowerment over time]*  **C3.1 Addressing social needs and motives**  **C3.2 Community empowerment and autonomy**  **C7 Innovation Embedding C7 (**e.g., through resource provision or regulations) [*Enabling resources and knowledge to be shared; Generalizing the process and methods and providing a holistic perspective; Changing the policies and regulations through community initiatives and active citizenships]*  **C7.1 Access to resources for transformative capacity building**  **C7.2 Planning and mainstreaming transformative action**  **C7.3 Reflexive and supportive regulatory frameworks**  **C10 Scale levels** (collective stewardship to enable and align diverse actions across political-administrative levels and geographical scales - site, neighbourhood, district, city, region, nation, inter- and transnational) *[Building networks across global and regional scales (a set of countries); Developing the capacity across national and state levels; Inspiring the capacity of the local governments*] | **C5.1 Diversity and transdisciplinary co-production of knowledge**  **C4 System(s) Awareness (**systems analysis to understand change dynamics and path dependencies) *[Strategic urban planning takes*  *a system’s perspective; Showcasing, advocating and gathering evidence on the performance of low-carbon solutions]*  **C4.1 Baseline analysis and system(s) awareness**  **C4.2 Recognition of path dependencies**  **C8 Reflexivity and Learning** (Social learning feeds outcomes of all four processes (C4-7) back into the articulation of agency i.e. governance,  leadership, and community empowerment (C1–3)) [*Critical discussions of the project development; Analysing and reporting the outcome of the projects; Evaluating and accessing the applied methods and tools]* |
| **(13) Grainger-Brown et al. (2022) on ‘Exploring urban transformation to inform the implementation of the Sustainable Development Goals’**  Identifies 15 ‘factors of urban transformation’ from a systematic literature review process, with the intent of using these to inform implementation of the SDGs. The factors can be viewed as a mix of mechanisms and enablers which are all identified in the literature as critical for urban transformation to occur or continue. | | | |
| Use of foresight tools and development of shared future narratives.  Strong local identity.  Experimentation across policy, capacity and actors.  Ongoing monitoring and data collection. | Open participation in the process.  Strong grassroots engagement and/or support.  Inter-stakeholder trust.  Openness towards innovation and change.  Collaboration and co-creation between different actors/ stakeholders. | Political institutional support.  Ongoing investment in the area.  Flexible regulatory/planning regime.    ‘Champion’ change agents/leaders.  Changes to community/ consumer behaviour. | Openness to learning and knowledge sharing between actors. |

### References

Abson DJ, Fischer J, Leventon J, Newig J, Schomerus T, Vilsmaier U, von Wehrden H, Abernethy P, Ives CD, Jager NW, Lang DL (2017) Leverage points for sustainability transformation. Ambio 46:30–39.

Beddoe R, Costanza R, Farley J, Garza E, Kent J, Kubiszewskia I, Martinez L, McCowen T, Murphy K, Myers N, Ogden Z, Stapleton K, Woodward J (2009) Overcoming systemic roadblocks to sustainability: The evolutionary redesign of worldviews, institutions, and technologies. Proceedings of the National Academy of Sciences 106:2483–2489.

Gorddard R, Colloff MJ, Wise RM, Ware D, Dunlop M (2016) Values, rules and knowledge: Adaptation as change in the decision context. Environmental Science & Policy 57:60–69.

Grainger-Brown J, Malekpour S, Raven R, Taylor E (2022) Exploring urban transformation to inform the implementation of the Sustainable Development Goals. Cities 131:103928. doi.org/10.1016/j.cities.2022.103928.

Grimm NB, Grove JM, Pickett STA, Redman CL (2000) Integrated approaches to long-term studies of urban ecological systems. BioScience 50:571–584.

Hölscher K, Frantzeskaki N, McPhearson T, Loorbach D (2019) Tales of transforming cities: Transformative climate governance capacities in New York City, U.S. and Rotterdam, Netherlands. Journal of Environmental Management 231:843–857.

Iwaniec D, Cook EM, Barbosa O, Grimm NB (2019) The framing of urban sustainability transformations. Sustainability 11(3):573. doi.org/10.3390/su11030573.

Kangas A, Kujala J, Lonnqvist A, Heikkinen A, Laihonen H (2019) Introduction: Leadership for dealing with complex changes. In: Kangas A, Kujala J, Heikkinen A, Lonqvist A, Laihonen H, [Bethwaite J (eds)](https://oapen.org/search?creator=Bethwaite,%20Julia) Leading change in a complex world: Transdisciplinary perspectives OAOPEN, The Hague, Netherands, pp 7-24.

Meadows D (1999) Leverage Points: Places to Intervene in a System. <https://donellameadows.org/archives/leverage-points-places-to-intervene-in-a-system/>. Accessed 16 October 2022.

Moser SC, Aldunce P, Rudnick A, Rojas M (2019) Transformations. Policy Brief for COP25, Santiago Chile, Madrid Spain.

Scoones I, Stirling A, Abrol D, Atela J, Charli-Joseph L, Eakin H, Ely A, Olsson P, Pereira L, Priya R, van Zwanenberg P, Yang L (2019) Transformations to sustainability: Combining structural, systemic and enabling approaches. Current Opinion in Environmental Sustainability 42:65–75.

Shahani F, Pinto-Pineta M, Frantzeskaki N (2021) Transformative low-carbon urban innovations: Operationalising transformative capacity for urban planning. Ambio 51:1179–1198. [doi.org/10.1007/s13280-021-01653-4](file:///C:\Users\bob\Documents\FEA%20SUD%20-%20National%20Strategy%20Article%20-%20submissions\UT%20V2%20submission\doi.org\10.1007\s13280-021-01653-4).

Waddell S (2016) Societal change systems: A framework to address wicked problems. The Journal of Applied Behavioural Science 52(4):422-449.

Wolfram M (2016) Conceptualising urban transformative capacity: A framework for research and policy. Cities 51:121–130.

Wolfram M, Borgstrom S, Farrelly M (2019) Urban transformative capacity: From concept to practice. Ambio 48:437–448. doi.org/10.1007/s13280-019-01169-y.
