# Supplementary material for: Enabling urban systems transformations: co-developing national and local strategies
Source: Urban Transform. 2023 Feb 20;5(1):5. doi: 10.1186/s42854-023-00049-9 (PMC9939254; doi:10.1186/s42854-023-00049-9)
Supplement: Supplementary file 4 — Additional file 4. Mapping of key themes in urban systems ‘science/knowledge/research’ articles to the KUST framework themes (KUST framework at Fig. 3 in main article text). [file 42854_2023_49_MOESM4_ESM.docx]

## Additional file 4 Mapping of key concepts in urban systems ‘science/knowledge/research themes’ articles to the KUST framework themes (KUST framework at Fig. 3 in main article text)

High level concepts from seven articles are included below in a tabular comparison with the Knowledge for Urban Systems Transformation (KUST) framework (Fig. 3 in the main article). All are about urban transformation knowledge and/or research framings. Some are framing research to date and others future research or knowledge priorities and/or frameworks. Several articles explicitly aim to advance a more convergent urban science, but have not previously been explicitly mapped together. It appears that each article views the urban as a complex adaptive system, and take a holistic systems approach, whether they are explicit on this or not. They are not intended to be an exhaustive list of such articles. Clearly there is a very large number of articles that look at more specific aspects of the urban systems from a number of ontological and epistemological positions, to focus on deeper understanding of specific issues. It may be helpful to understand how these might enrich or challenge the framework. However, our interest here is in those who have attempted a more holistic framing of urban knowledge.

Comparison shows that the concepts can be mapped quite well to components of the KUST framework (and therefore to each other). The mapping is not always exact as each source has its own emphasis, and may use somewhat different terminology. However, it appears they are all describing essentially the same high-level urban knowledge paradigm.

The intent is not to take away from the independent value of each published article – there is clear value in the different perspectives, and each explores significant detail within individual concepts reflecting its own focus. In fact, reflecting the intrinsic complexity of the urban systems, the comparison shows that there are many ways that an urban challenge and research program/project can be validly structured. This is often by entering the complexity through a particular thematic window, and then drawing on various additional themes to reflect aspects of the complex urban system. The challenge in practical use is to capture the most relevant themes for the purpose at hand.

The studies should therefore be seen as complementary rather than competing views.

However, at the same time, in order to make research more convergent, cumulative and useful to policy-makers and practitioners, there is also value in showing how they can be related to each other, with the extra confidence that they can also be matched to a common framework such as the KUST. It also shows that the KUST framework, derived primarily from a transdisciplinary engagement process, is quite robust.

### Fig. 3 Knowledge for Urban Systems Transformation (KUST) Framework

The transdisciplinary process participants focused on themes that could directly influence future outcomes (via collaborative direction-setting, governance/decision-making and enabling processes: Theme A); supported by knowledge to overcome static views of urban systems, and policy, practice and research siloes (via broader, interconnected and dynamic urban systems view: Themes B, C, D), and access to enhanced knowledge system capabilities (Theme E). Those involved in Theme A processes need to recognise they are themselves also part of the urban systems subject to change (e.g. in Social and Institutional process sub-themes: Theme B2). (See also “Systems-wide frameworks as boundary objects” section in main article “Discussion” for KUST connection to other recent studies).

### Table Af4 Mapping between the ‘KUST’ framework and urban science/research/knowledge themes from other studies

| **Mapping between the ‘KUST’ framework and urban science/research/knowledge themes from other studies** | | |
| --- | --- | --- |
| **Urban science/research/knowledge article (source)** | **Key concepts from source article**  **(mapped to KUST framework themes in parentheses: see Fig. 3 above for KUST themes coding)** | **KUST framework equivalent concepts**  **(see Fig. 3 above for KUST themes coding)** |
|  |  |  |
| **(1) Zhou et al. (2021) ‘Conceptual frameworks facilitate integration for transdisciplinary urban science’**  Proposes nested conceptual frameworks to support and operationalise a convergent SETS-based urban science | Urban understood as SETS  **Four overarching global urban conditions:**  Complexity, diffuseness (porous across scales), connectedness, diversity  **Five nested frameworks**: (e.g. useful to test framing as a checklist of sequential questions)  The human ecosystem i.e. SETS (A-E)  Disturbances (A, C, D)  Resilience (B)  Dynamic heterogeneity (B)  Continuum of urbanity (modes and effects of connectivity locally and distant; all locations a mix of urban and rural/wild); each location characterised by livelihood, lifestyle, connection, SETS structure/perception (B, B1, B2)  **Metacity** is a conceptual device that needs the 5 above frameworks to create operational multi-scalar urban models. It is represented as an urban mosaic, at any scale, that consists of patches differentiated by their hybrid structures and interacting SETS processes and flows of energy, organisms (including people), information, and materials  Consequently, a metacity is a comprehensive way to represent the spatial, temporal, structural, and process-based features of any urban system. (A, B, C, D) | Urban understood as SETS  Urban is complex, connected across scales and processes, diverse, dynamic/adaptive  A-E: Urban systems as SETS  A, C, D: Systems subject to disturbances (governance/social decisions A; opportunities/ innovation C; and risks/pressures/crises D)  B: Change impact subject to systems resilience  B: Dynamic, emergent and heterogeneous change  B, B1, B2: Connectedness between multiple sectors/scales by flows of people, information, money, energy, materials, products, organisms (B2.1-4); at locations local to international with urban to rural to remote continuum (B1.1-4); each location characterised by emergent and heterogeneous SETS outcomes.  A, B, C, D: This is an alternative but similar way of describing the interactions of the system themes. The application of KUST for a specific location, and its spatial and process interactions, will always be intensely context specific. The KUST framework can be seen as complementary as the Metacity mosaic and patch description very well evokes the spatial and process multi-scalar heterogeneity (using the well-developed urban ecological concept of mosaics and patches), and KUST provides more ground-up articulation of some of the SETS spatial and process components (Themes B1 and B2), and of the view into the system from the perspective of those aiming to collaboratively progress the transformations (via Theme A) |
|  |  |  |
| **(2) Hölscher and Frantzeskaki (2021) ‘Perspectives on urban transformation research: transformations in, of, and by cities’**  Proposes three perspectives on urban transformations to structure and guide research and practice; the perspectives also outline an agenda for advancing theory and practice for urban sustainability and resilience. | Cities are understood as complex, adaptive and open SETS.  **Three perspectives on urban transformation research:** look at systemic change dynamics taking place in cities (“in”), the outcomes of systemic change of cities (“of”), or systemic change on global and regional levels driven by cities (“by”):  In cities: cities as spaces. (B1.1-2 view into rest of A, B, C, D)  Of cities: urban (sub-)systems defined by specific functions (B1.2 view into rest of A, B, C, D)   - By cities: networks of global resource flows, commodities, communication and governance; cities as “agents of change at global scale”; supports a polycentric and multi-level approach to global environmental governance. (B1.2-4 view into rest of A, B, C, D)   A future research direction put forth is to bridge across the three perspectives to address their respective limitations and generate comprehensive actionable knowledge. | KUST based on complex, adaptive/resilient, open urban SETS.  The three perspectives could be seen as viewing the complex urban systems (A, B2, C, D) and cross-scale interactions (B1) through three spatial lenses:  B1.1-2: at household to city/region scale  B1.2: at city/region scale  B1.2-4: at city/region-national-international scale (with both ways flows and impacts)  An agenda to progressively integrate across A, B, C, D in a particular context |
|  |  |  |
| **(3) Frantzeskaki, McPhearson and Kabisch (2021) ‘Urban sustainability science: prospects for innovations through a system’s perspective, relational and transformations’ approaches’**  Proposes three perspectives/approaches into SETS urban science based on past AMBIO research publications, that also should guide and shape future research pathways | Urban understood as SETS  **Three innovation pathways in urban sustainability science**:   - Systems perspective: requires integrative solutions to work in the dynamic tripled social -ecological-technological system (SETS) setting. Including sustainability and policy assessment frameworks, from local to planetary scale. (A1, B, C, D) - (People and place) relational perspective: Urban sustainability as a contested and an ever changing social-ecological contract of cities. Need to understand personal and agency motivations, including social and environmental justice; and what constitutes actionable knowledge. Requires coordinated action to bridge people, places, meanings, visions and ecosystems. (A2/B2.4, A3, B1.1-2) - Transformation approach: for urban sustainability to be achieved and progressed, deep transformations are required in systems, relations, policies and governance approaches. Need to progress research on experimentation research, systematization of co-creation and co-production approaches to urban sustainability and to innovative bridging concepts and frameworks, e.g. transformative capacity (A1, A2, A3)   Next to the three proposed research pathways, also see the benefits that a critical synthesis of knowledge within and across those pathways can yield, such as insights about depths of knowledge and blind spots that require new conceptualization and empirical investigation | Urban understood as SETS  Can be seen as three complementary windows into the urban SETS  B, C, D: Dynamic SETS perspective.  A1: Sustainable development outcomes assessment based on policy and other drivers  B1.1-4 From local to planetary scale  A2/B2.4, A3, B1.1-2: Relational governance (A2/B2.4) including through understanding, contestation and negotiation of visions, values and behaviours (motivations), and insights from knowledge (part of A3 enabling capacities); related to local to city scales where relational approach most relevant (i.e. B1.1-2)  A1, A2, A3: Transformation direction and navigation research (A1), supported by research on governance processes (A2) and an enabling capacities framework (EUST A3) The latter includes research on co-designed and co-developed experimentation, engagement approaches, governance and systems knowledge topics, and aims to help bridge from desired outcomes to implementation.  Requires integration across A, B, C, D, including across the interdependent components of A3 (EUST framework) |
|  |  |  |
| **(4) Prieur-Richard et al. (2019) ‘Global Research and Action Agenda on Cities and Climate Change Science’**  Developed from a 2018 Edmonton Conference, jointly sponsored with IPCC, proposes a Research and Action Agenda for future research into climate change and cities | Cities as systems operating across spatial and temporal scales  **Four cross cutting issues and knowledge gaps:**   - Systems approach (A, B, C, D) - Spatial and temporal scale (B, B1)      - Observations, data, modelling, and scenarios at the city level (E1) - Governance and institutions (A2/B2.4)   **Six key topical knowledge gaps**:   - Urban planning and design (A2/ B2.4) - Sustainable consumption & production (B2.1-3) - Built & blue/green infrastructure (B2.3) - Informality (B1.1, B2.4) - Finance (A2, A3) - Uncertainty (understanding sources and decision-making) (A2, B, C, D)   **Three enablers to deliver on the Research and Action Agenda**: Approaches to strengthen the science, practice and policy interface   - Empowering cities to take action (A3) - Fostering long-term science-policy-practice partnerships (A3) - Knowledge co-design and co-production (A3) | Cities as complex adaptive SETS  A, B, C, D: Systems approach including disturbances  B, B1: At various spatial (B1) and temporal (B dynamic SETS) scales  E1: Knowledge systems include models and data  A2/B2.4: Governance and institutional processes  A2/B2.4: Governance includes planning and design  B2.1-3: Social, environmental and technological processes all include SC&P potential  B2.3: Includes built and BGI infrastructure  B1.1: Includes place-based informal settlements  B2.4: Includes informal economies  A2, A3: Governance processes (A2) and enabling capacities (A3) include planning and finance  A2, B, C, D: Uncertainty comes from complex emergent SETS including under disturbances (B, C, D); approaches to decision-making under uncertainty is part of governance processes (A2)  A3: provides a comprehensive set of transformation enabling capacities  A3: EUST framework includes empowering at all levels from cities through to community groups (EUST capacity 3.4); policy-practice-research collaboration (EUST capacity 4.4); and co-design and co-production of knowledge (EUST capacity 4.1) |
|  |  |  |
| **(5) Ramaswami et al. (2018) ‘Sustainable Urban Systems: Articulating a Long-Term Convergence Research Agenda.’ (Report for ACERE)**  This report for the US National Science Foundation’s ACERE, articulates a vision and a research agenda for developing the next generation of sustainable urban systems (SUS) science | **Urban systems are geographical areas with a high concentration of human activity and interactions, embedded within multiscale interdependent social, engineered, and natural systems that impact human and planetary well-being across spatial (local to global) and temporal scales.**  **3 perspectives:** Advancing the next generation of SUS science requires intentional integration across three perspectives:   - Single urban areas/metropolitan regions – multi-scale systems perspective (B1, B1.2) - Multiple cities and communities – typologies and comparisons, networks (B1.1-2, E2.1) - Supra-aggregation of cities – nation, world region, world, collective impact (B1.3-4)   **6 key elements**: Within each of the three perspectives integration of the following six key elements is expected to significantly advance SUS science.   - Natural, human-built, and social systems and associated data and methods (B/C/D, E1) - Sustainability outcomes nexus (A1, B) - Levers for (theories of) change (A1, A2/B2.4, A3)   - design, innovation, STS   - multilevel actor and governance - Comparative scalability studies to generalize theories of change (A1, A2/B2.4, E1) - Modeling future of SUS across 3 perspectives (see above) (A, B/C/D, E1) - Knowledge coproduction and real-world experimentation (A3, E2) | **Urban understood as SETS operating across spatial and temporal scales**  B1: Spatial view of the urban systems  B1, B1.2: City/region scale (B1.2) but connected by systems and processes across scales (B1)  B1.1-2, E2.1: Knowledge infrastructure (E2.1) includes comparative methodologies including urban typologies facilitating comparative analysis across cities (B1.2) and communities (B1.1)  B1.3-4: National and international scale impacts of cities (B1.3 to B1.4 - national to global)  B/C/D, E1: Urban systems as SETS (B/C/D), associated data and methods (E1)  A1, B: Intended urban outcomes (A1), supported by systems and outcomes nexus understanding (B)  A1, A2/B2.4, A3: Governance processes (A2/B2.4) include ‘theories of change’, linking intended outcomes (A1) to determinants/levers of outcomes including design and innovation, and multi-level actors and governance (parts of A3)  A1, A2/B2.4, A3, E1: Knowledge infrastructure (E1 comparative typology and scalability methodology) supports generalised conclusions that can be applied to understand transformation outcomes and determinants (A1, A2/B2.4, A3)  A, B/C/D, E1: Future modelling (E1) of urban systems and interventions/disturbances (B/C/D) and outcomes (A1) across 3 spatial perspectives (see above)  A3, E2: Knowledge processes (E2 co-production) and co-production and experimentation capacities (A3 EUST framework capacities 4.1, 1.3) |
|  |  |  |
| **(6) Wolfram, Frantzeskaki and Maschmeyer (2017) ‘Cities, systems and sustainability: status and perspectives of research on urban transformations.**  This paper identifies and maps out the current status of research on urban transformations, noting STS- and SES-based studies in particular, and derives strategic recommendations for future research themes to exploit potential synergies and address gaps | STS and SES orientation  Seven key factors co-shaping urban transformations are recognised (agency, political power, stakeholder capacity, policy/planning, experiments, foresight and geography). (B1, A3)  Current status: Specific epistemological orientations are derived in particular from STS and SES studies, since most scholars focus on one of the following four issues:     - Urban metabolism and political ecologies, powerful urban actors, infrastructure and technology providers (STS-based) (A2/B2.4, B1, B2.3/C) - Resilient communities and ecosystems, ecosystem services, blue/green infrastructure, diverse actors (SES-based) (A2/B2.4, B1.1-2, B2.1-2, C/D) - Urban grassroots and social innovations, heterogeneous, civil society actors, local place-based (STS-based) (A2/B2.4, B1.1/B2.1/C, B2.1) - Urban innovation systems for green economies, private sector, consumers, markets, sustainable consumption and production, infrastructure (STS-based) (A2/B2.4, B1, B2.1-3)   Future themes: To better exploit potential synergies between existing strands and address gaps in the light of imminent urban sustainability challenges, future urban transformation research should:  Share a relational geographical perspective that connects the above epistemologies across spatial scales, systems dynamics, and the role of policy, planning and politics (A/B/C/D, B1)  Identify and engage with the spatial institutional challenges of urban transformations (e.g., urban/rural dependencies, metropolitan areas, cross-border urban development or urban tele-connections), especially politics and power (A2/B2.4, B1)  Move towards multi-system or nexus approaches linking various sectors (e.g. energy, water, waste, transport) and domains (e.g. land-use, urban form, biodiversity, food or health) (B, B1, B2)  Focus on transformative capacity and its agency components (empowered communities, transformative leadership, inclusive collective action, etc.) as an empowering lever for systemic urban change (A3) | Urban as SETS  B1, A3: First six of these factors are reflected in enabling capacities (A3). Geography is reflected in spatial view (B1).  A2/B2.4, B1, B2.3/C: urban metabolism/ technology innovation (B2.3/C); political ecologies and economies, power, actors (A2/B2.4) potentially across all spatial scales (B1)  A2/B2.4, B1.1-2, B2.1-2, C/D: ecosystem and social processes (B2.1-2) at local-city scale (B1.1-2) (including innovative opportunities C and resilience to stress and shock disturbances D); diverse actors in decision making processes (A2/B2.4)  A2/B2.4, B1.1/B2.1/C social innovations (B2.1/C) at place based neighbourhood to building scale B1.1); civil society agency (A2/B2.4)  A2/B2.4, B2.1-3 Green economies including SC&P (B2.1-3); private sector/consumer market driven (A2/B2.4); can be at multiple spatial scales (B1)  A/B/C/D, B1: spatial geography perspective connected across scales (B1), relations linked through to urban systems dynamics processes (A/B/C/D), including policy, planning and politics (A2/B2.4)  A2/B2.4, B1: institutional processes, including politics and power (A2/B2.4) playing out across all spatial scales (B1)  B, B1, B2: multi sector/domain/nexus/outcome systems (B2) including nexus approaches (B); potentially across all scales (B1)  A3: Urban systems enabling capacities (A3/ EUST framework) |
|  |  |  |
| **(7) Wolfram and Frantzeskaki (2016) ‘Cities and Systemic Change for Sustainability: Prevailing Epistemologies and an Emerging Research Agenda’**  Recognises four current and salient urban research epistemologies and proposes four themes for future research | Combines systemic studies (STS, SES perspectives) with urban studies (a relational geography perspective with cities as socially-constructed nodes (i.e. with potentially competing visions from actors at all scales), in ‘SETS’ networks with flows of people, matter and information across scales; this perspective supported by several disciplines e.g. urban economic geography, political ecology, planning studies, sociology); and a number of change drivers.  Recognises four salient research  epistemologies, each focusing on a distinct combination of drivers of change:   - transforming urban metabolisms and political ecologies - building adaptive urban communities and ecosystems - empowering urban grassroots niches and social innovation - configuring urban innovation systems for green economies   The findings suggest that future research directed at cities and systemic change towards sustainability should:   - explore using relational geography and multi-level governance theory as boundary areas to frame spatial, institutional and temporal systemic changes, and investigate how the change drivers impact on the urban systems over time including tipping points (A2/B2.4, B1, B2) - consistent with the above, conceive of cities as places shaped by and shaping interactions between multiple socio-technical and social-ecological systems i.e. as SETS, understanding how institutions and actors approach the reality and implications for systems and outcomes in particular urban locations, also accounting for their trans-local and scalar relations (A2/B2.4, B1, B/C/D) - focus on agency across systems and drivers of change, and develop corresponding approaches for intervention and experimentation, including new forms of urban governance, intermediation and institutional entrepreneurship (A2/B2.4, A3, B1) - rebalance the empirical basis and methods employed, strengthening transdisciplinarity and comparative studies across case studies in particular (E1, E2) | Urban systems as spatially connected SETS networks and flows (B) subject to internal and external change drivers (A/C/D); with cities as nodes that are socially constructed at local-national and increasingly international scales (B1, B2.1)  See Wolfram et al. 2017 above, as effectively the same four to-date epistemologies are identified  A/B/C/D, A2/B2.4, B1, B2: spatial and relational geography perspective across scales (B1) and institutional/governance processes (A2/B2.4) to investigate drivers (A/C/D) impact on urban systems over time (B)  A2/B4, B1, B/C/D: urban seen as SETS (B/C/D), subject to institutional and other actors’ actions (A2/B2.4), with specifics subject to local and trans-local relations (B1)  A2/B2.4, A3, B1: formal and informal agency in urban decisions (A2/B2.4); building current and new governance and institutional (including community) capacities (A3) potentially across all scales  E1, E2 Knowledge process include transdisciplinary co-production (E2), and knowledge infrastructure includes comparative studies and urban typologies (E1) |
|  |  |  |

### References

Frantzeskaki N, McPhearson T, Kabisch N (2021) Urban sustainability science: Prospects for innovations through a system’s perspective, relational and transformations’ approaches. Ambio 50:1650-1658. doi.org/10.1007/s13280-021-01521-1.

Hölscher K, Frantzeskaki N (2021) Perspectives on urban transformation research: Transformations in, of, and by cities. Urban Transformations 3:2. doi.org/10.1186/s42854-021-00019-z.

Prieur-Richard AH, Walsh B, Craig M, Melamed ML, Colbert M, Pathak M, Connors S, Bai X, Barau A, Bulkeley H, Cleugh H, Cohen M, Colenbrander S, Dodman D, Dhakal S, Dawson R, Espey J, Greenwalt J, Kurian P, Lee B, Leonardsen L, Masson-Delmotte V, Munshi D, Okem A, Delgado Ramos GC, Sanchez Rodriguez R, Roberts D, Rosenzweig C, Schultz S, Seto K, Solecki W, van Staden M, Ürge-Vorsatz D (2019) Global research and action agenda on cities and climate change science - Full Version. World Climate Research Programme, Publication No.13/2019, 31pp. https://www.wcrp-climate.org/news/wcrp-news/1517-graa-published. Accessed 15 December 2021.

Ramaswami A, Bettencourt L, Clarens A, Das S, Fitzgerald G, Irwin E, Pataki D, Pincetl S, Seto K, Waddell P (2018) Sustainable urban systems: Articulating a long-term convergence research agenda. Report of the Advisory Committee for Environmental Research and Education (ACERE), Vol 31. The National Science Foundation. https://www.nsf.gov/ere/ereweb/ac-ere/sustainable-urbansystems.pdf. Accessed 15 December 2021.

Wolfram M, Frantzeskaki N (2016) Cities and systemic change for sustainability: Prevailing epistemologies and an emerging research agenda. Sustainability 8:144. doi.org/10.3390/su8020144.

Wolfram M, Frantzeskaki N, Maschmeyer S (2017) Cities, systems and sustainability: Status and perspectives of research on urban transformations. Current Opinion in Environmental Sustainability 22:18–25.

Zhou W, Pickett STA, McPhearson T (2021) Conceptual frameworks facilitate integration for transdisciplinary urban science. Npj Urban Sustainability 1(1):1–11. [doi.org/10.1038/s42949-020-00011-9](file:///C:\Users\bob\Documents\FEA%20SUD%20-%20National%20Strategy%20Article%20-%20submissions\UT%20V2%20submission\doi.org\10.1038\s42949-020-00011-9).
